# Supplementary material for: Structural basis for the ligand recognition and signaling of free fatty acid receptors
Source: Sci Adv. 2024 Jan 10;10(2):eadj2384. doi: 10.1126/sciadv.adj2384 (PMC10780892; doi:10.1126/sciadv.adj2384)
Supplement: Supplementary file 1 — Supplemental Methods Figs. S1 to S16 Tables S1 to S4 References [file sciadv.adj2384_sm.pdf]

Science Advances
AAAS

Supplementary Materials for

Structural basis for the ligand recognition and signaling of free fatty acid receptors

Xuan Zhang *et al.*

Corresponding author: Cheng Zhang, chengzh@pitt.edu; Irina G. Tikhonova, i.tikhonova@qub.ac.uk; Graeme Milligan graeme.milligan@glasgow.ac.uk

Sci. Adv. **10**, eadj2384 (2024)
DOI: 10.1126/sciadv.adj2384

This PDF file includes:

Supplemental Methods
Figs. S1 to S16
Tables S1 to S4
References

Supplemental Methods

Phylogenetic analysis

Phylogenetic analysis of full-length sequences of human class A GPCRs (312 in total) was done using the sequences and tools provided by GPCRdb web server [97]. Unrooted phylogenetic tree with increasing node order was built using FigTree v1.4.4 [98]. All GPCRs were colored according to their GPCRdb ligand type. Nodes with descendants sharing the same GPCRdb ligand type were colored accordingly, with the remaining nodes colored grey.

Molecular Docking

The protein structures were prepared with the protein preparation module, and the structure of TUG-1197 was assessed with the ligand preparation module of Schrodinger software. TUG-891 from the FFA4-TUG-891 complex was selected as the center of the docking box. Receptor docking grids were generated with the receptor van der Waals radius scaling of 1.0. Docking poses were obtained and evaluated with the Glide program [99-102]. The OPLS_2005 force field was used in all calculations.

MD simulations

All molecules except the GPCR and, if necessary, the ligand and G-protein, were removed using Schrodinger Maestro 2021-3. Missing loops and sidechain atoms of the proteins were filled using knowledge-based homology modelling of the Prime module [103-105] with amino acid sequences taken from the UniProt database [106] for all protein chains except the engineered miniGq subunit, for which the sequence was taken from the Protein Data Bank. The N- and C- termini of the receptors were extended by up to 3 residues as per the UniProt sequence, and the added residues were minimized using the 3D builder of Maestro. The N- and C-termini of the receptors and the G-protein were capped with acetyl and N-methyl groups, respectively. The obtained structures were analyzed using the Maestro Protein Reports tool and strong steric clashes were removed by local geometry minimization.

The protonation states of amino acids at pH 7.4 were predicted by PROPKA 3 as a separate application [107,108] and as implemented in the Maestro Protein Preparation workflow. Thus, $D208^{5.39}$ in the FFA4-TUG-891 complex and $H140^{4.56}$ in the FFA2-butyrate complex were kept protonated in the simulations. All non-protonated histidine residues were taken as a δ-tautomer except $H242^{6.55}$ in the FFA2-butyrate complex, which was taken as an ε-tautomer as it reduced the root mean square deviation of the ligand atoms.

The receptor was oriented in membrane using the PPM server within CHARMM-GUI [109]. The ligand parameter files were created by the Antechamber utility of the CHARMM-GUI server with the AM1BCC charge scheme and GAFF2 atom types.

The receptor was placed in the 1-palmitoyl-2-oleoyl-sn-glycero-3-phosphocholine (POPC) bilayer membrane sized 100 Å x 100 Å and 22.5 Å solvent layer on each side of the membrane. For receptor-miniGq systems, 120 Å x 120 Å membrane bilayer was used. The solvent contained 150

mM NaCl, and the total number of atoms was between 100,000 and 130,000 atoms for receptor-only systems and between 240,000 and 260,000 atoms for the receptor-miniGq systems.

The minimization, equilibration and production were done using the PMEMD program from the Amber20 package [89-91] using the ff19SB [110], LIPID21 [111], GAFF2 [112] force fields and the TIP3P model [113] for the protein, membrane lipids, ligands and water, respectively. The nonbonded interaction cut-off was set as 9 Å.

As per the recommended CHARMM-GUI protocol, the initial energy minimization included 2500 steepest descent steps followed by 2500 more steps using the conjugated gradient method. The equilibration steps followed the same pattern of gradually decreasing the force constants of positional restraints but were conducted with longer simulation length than recommended by the CHARMM-GUI protocol. Heating to 310 K was done in the NVT ensemble with 1 fs timestep in two consecutive equilibration steps, each 2.5 ns long. This was followed by a 2.5 ns NPT equilibration with Berendsen barostat with a τ_{coupl} of 1.0 ps^{-1} and a 1 fs timestep [114]. The next two steps of NPT equilibration used 2 fs timestep and lasted for 10 ns each, and the final step in which only the protein backbone was restrained lasted 20 ns. For equilibration and production steps, Langevin thermostat [115] with a friction coefficient of 1.0 ps^{-1} was used. The production was done with Berendsen barostat with τ_{coupl} of 1.0 ps^{-1} and a timestep of 2 fs.

Supplemental figures

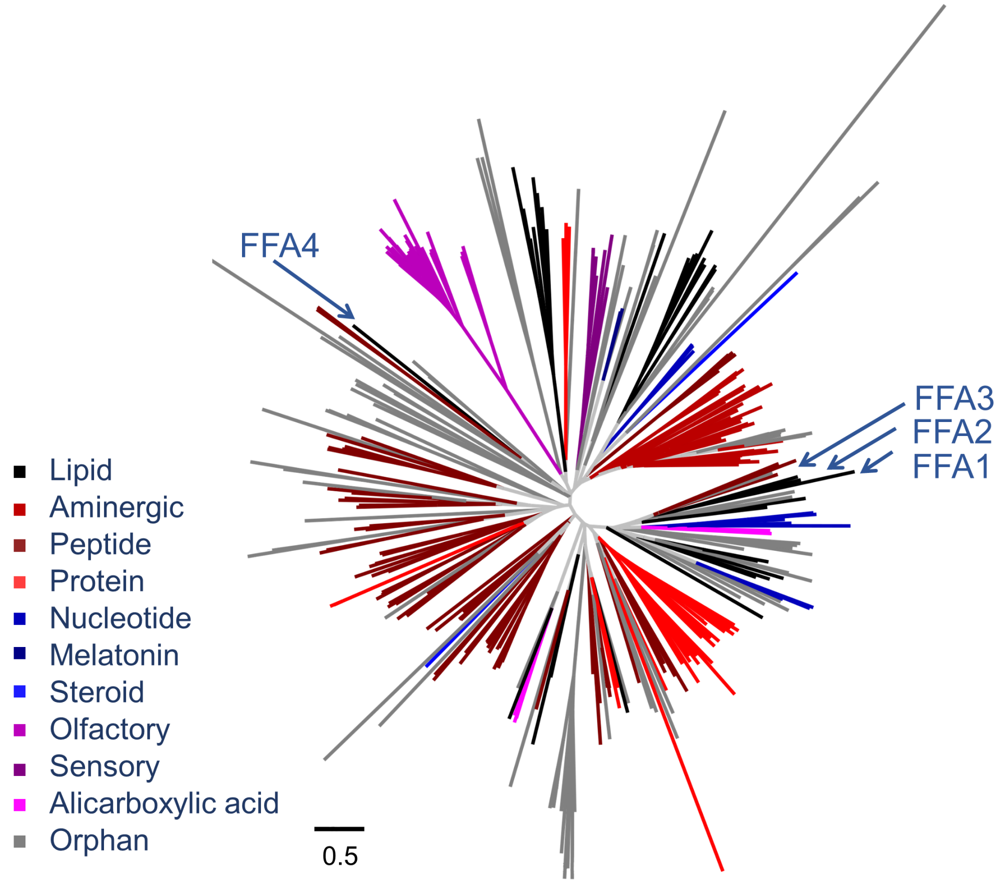

Figure S1. Phylogenetic analysis of the FFA receptor family. Clustering of full-length sequences of human class A GPCRs (312 in total) was done using the sequences and tools provided by the GPCRdb web server (https://gpcrdb.org/). An unrooted phylogenetic tree with an increasing node order was built using FigTree v1.4.4 (http://tree.bio.ed.ac.uk/). All GPCRs were colored according to their GPCRdb ligand type. Nodes with descendants sharing the same GPCRdb ligand type were colored accordingly, with the remaining nodes colored grey.

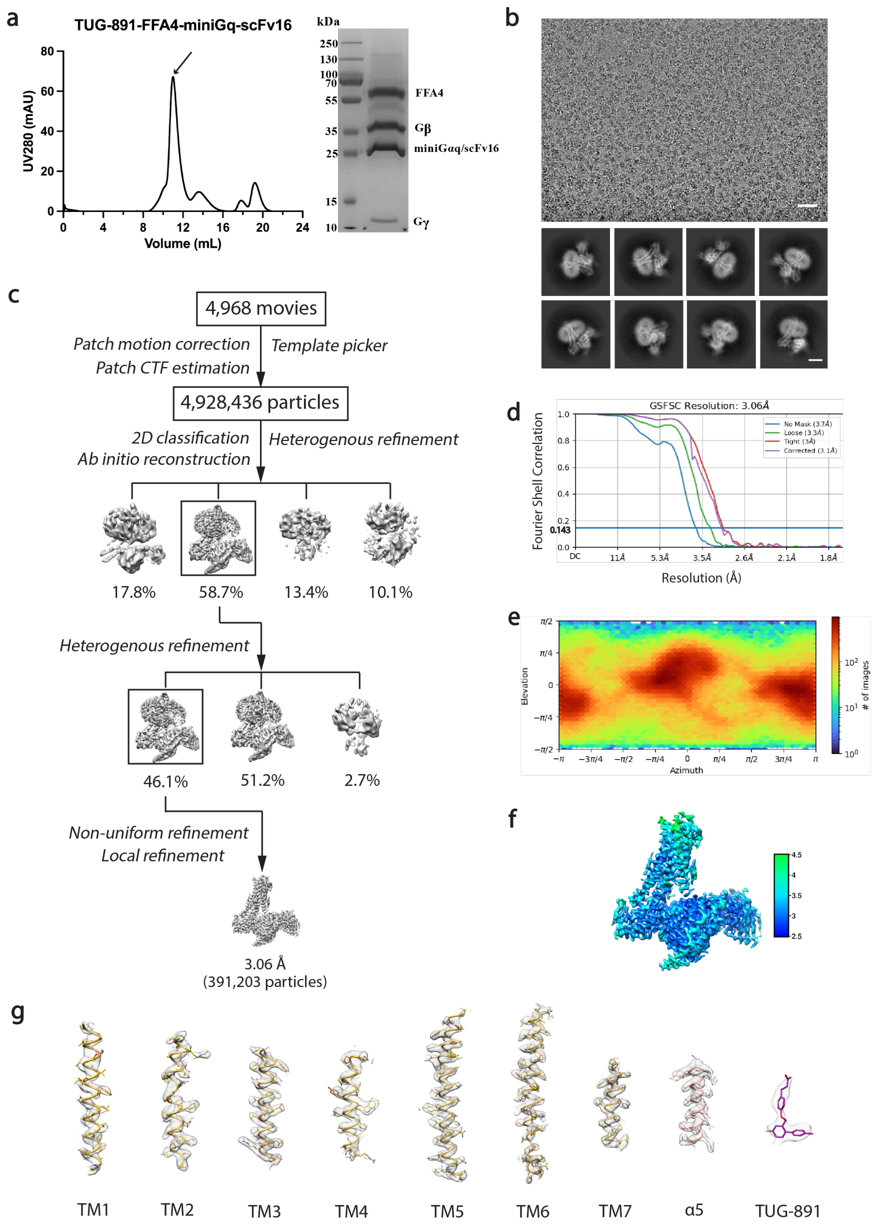

Figure S2. Purification of the FFA4-miniGq complex with TUG-891 and cryo-EM data processing. (a) Size-exclusion chromatography profile and SDS-PAGE analysis of the purified TUG-891-FFA4-miniGq complex. **(b)** Representative cryo-EM micrograph (scale bar: 50 nm) and 2D class averages (scale bar: 5 nm). **(c)** Cryo-EM image processing workflow for the TUG-891-FFA4-miniGq complex. **(d)** Gold-standard Fourier shell correlation (FSC) curve showing an overall resolution is 3.06 Å at FSC=0.143. **(e)** Angular distribution of the particles used in the final reconstruction. **(f)** Density map according to local resolution estimation. **(g)** Cryo-EM density maps and models of the seven transmembrane helices (TM1-7), α5 helix of miniGq and the ligand of TUG-891 bound FFA4-miniGq complex are shown. The EM density is shown at 0.147 threshold.

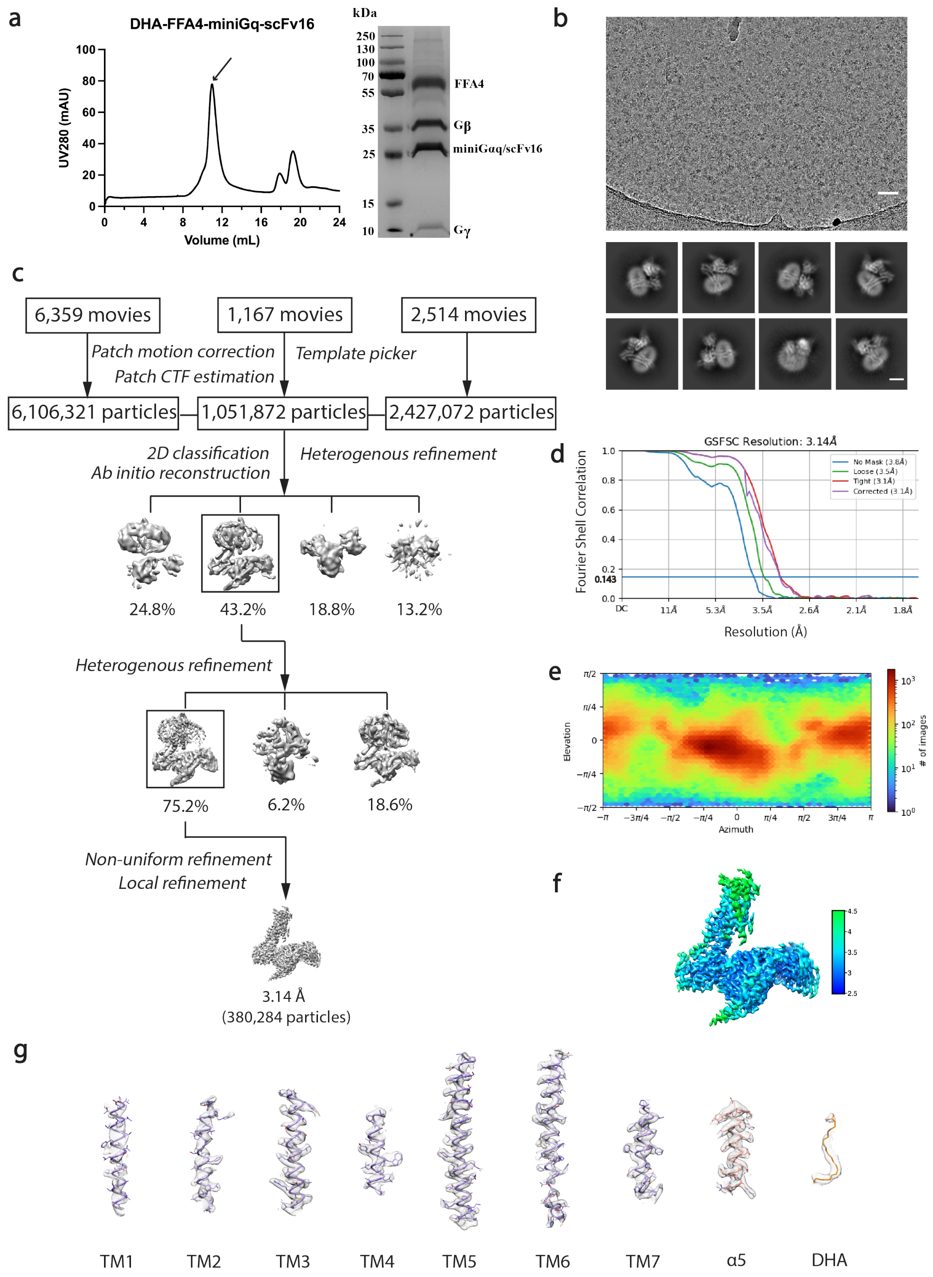

Figure S3. Purification of the FFA4-miniGq complex with DHA and cryo-EM data processing. (a) Size-exclusion chromatography profile and SDS-PAGE analysis of the purified DHA-FFA4-miniGq complex. **(b)** Representative cryo-EM micrograph (scale bar: 50 nm) and 2D class averages (scale bar: 5 nm). **(c)** Cryo-EM image processing workflow for the DHA-FFA4-miniGq complex. **(d)** Gold-standard Fourier shell correlation (FSC) curve showing an overall resolution is 3.14 Å at FSC=0.143. **(e)** Angular distribution of the particles used in the final reconstruction. **(f)** Density map according to local resolution estimation. **(g)** Cryo-EM density maps and models of the seven transmembrane helices (TM1-7), α5 helix of miniGq and the ligand of DHA bound FFA4-miniGq complex are shown. The EM density is shown at 0.132 threshold.

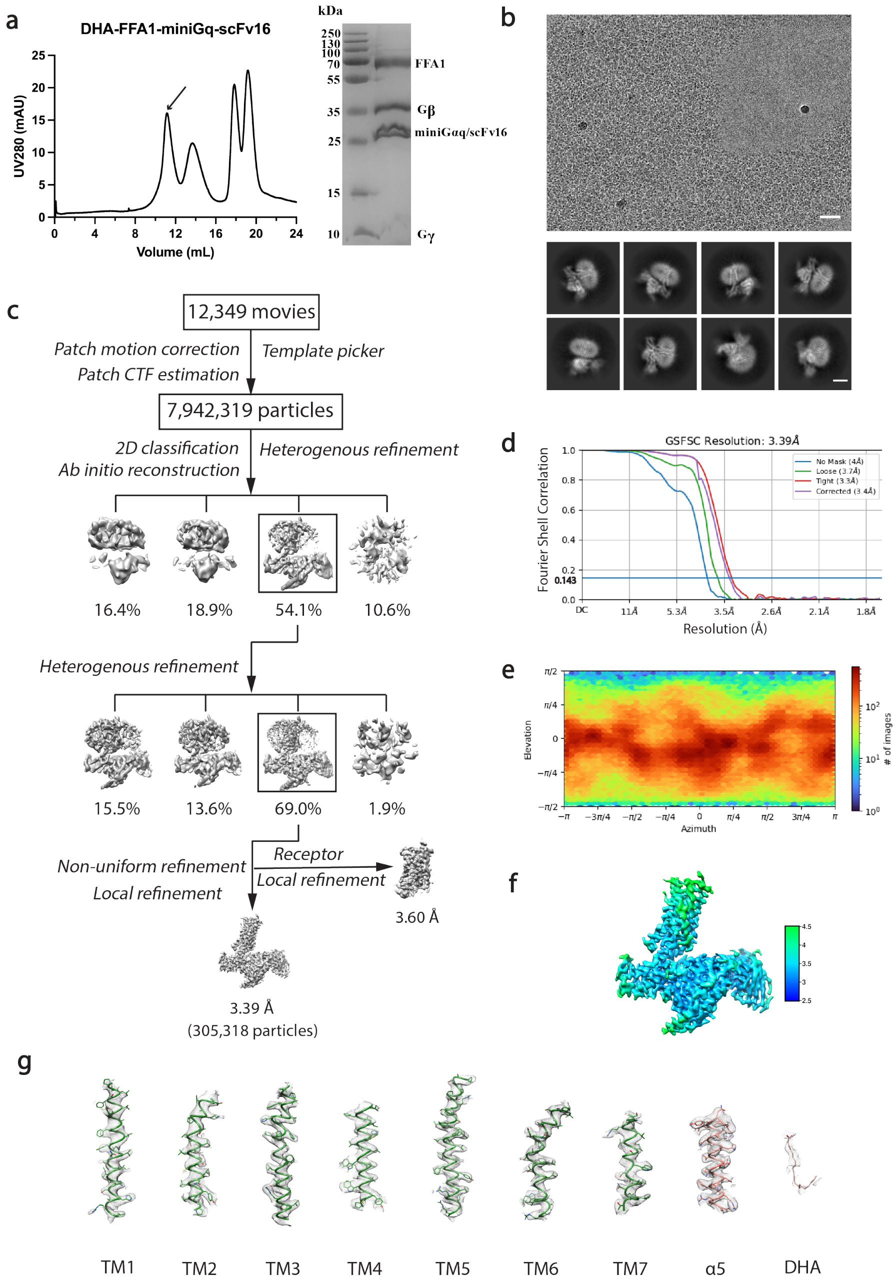

Figure S4. Purification of the FFA1-miniGq complex with DHA and cryo-EM data processing. (a) Size-exclusion chromatography profile and SDS-PAGE analysis of the purified DHA-FFA1-miniGq complex. **(b)** Representative cryo-EM micrograph (scale bar: 50 nm) and 2D class averages (scale bar: 5 nm). **(c)** Cryo-EM image processing workflow for the DHA-FFA1-miniGq complex. **(d)** Gold-standard Fourier shell correlation (FSC) curve showing an overall resolution is 3.39 Å at FSC=0.143. **(e)** Angular distribution of the particles used in the final reconstruction. **(f)** Density map according to local resolution estimation. **(g)** Cryo-EM density maps and models of the seven transmembrane helices (TM1-7), α5 helix of miniGq and the ligand of DHA bound FFA1-miniGq complex are shown. The EM density is shown at 0.128 threshold.

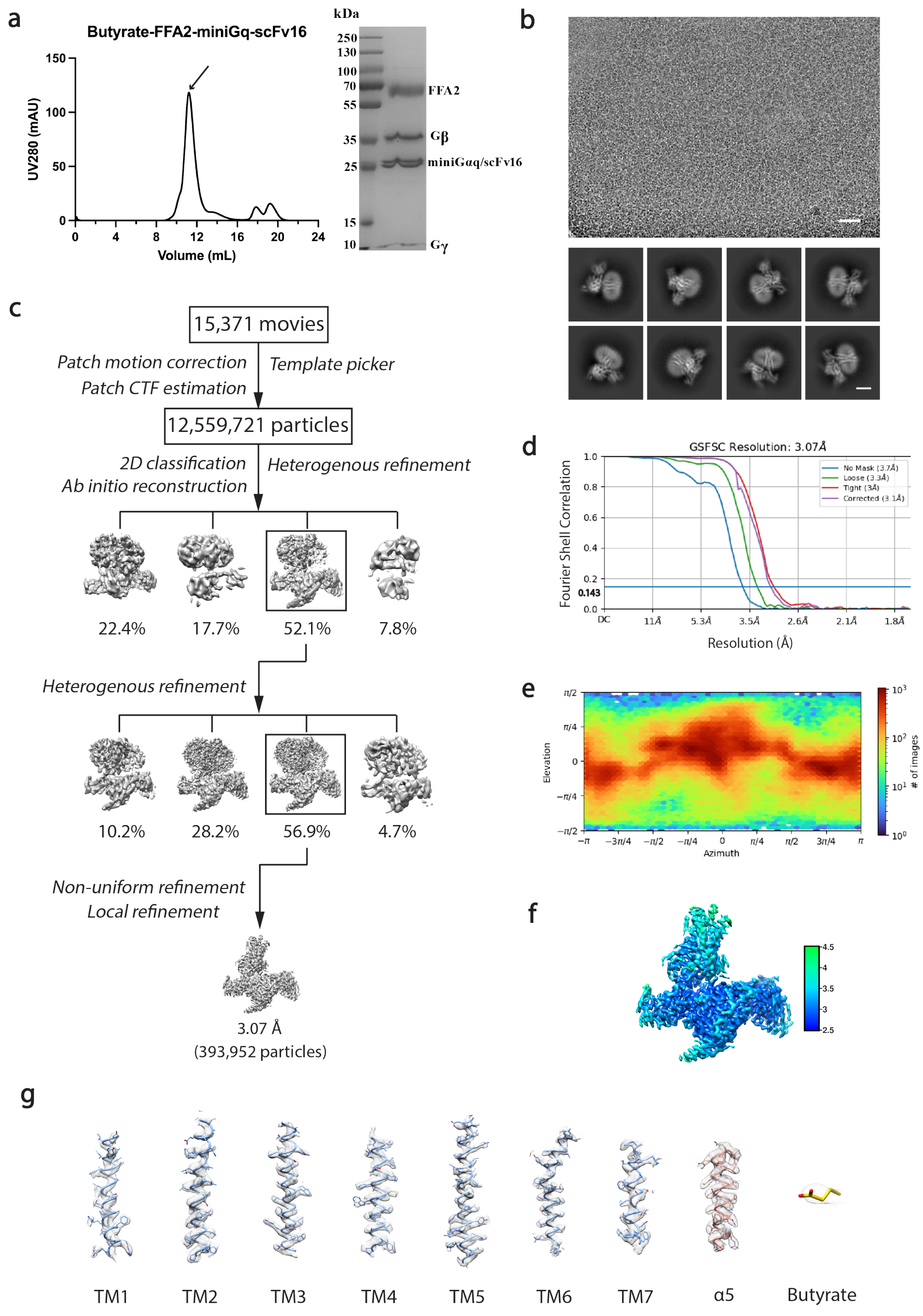

Figure S5. Purification of the FFA2-miniGq complex with Butyrate and cryo-EM data processing. (a) Size-exclusion chromatography profile and SDS-PAGE analysis of the purified Butyrate-FFA2-miniGq complex. **(b)** Representative cryo-EM micrograph (scale bar: 50 nm) and 2D class averages (scale bar: 5 nm). **(c)** Cryo-EM image processing workflow for the Butyrate-FFA2-miniGq complex. **(d)** Gold-standard Fourier shell correlation (FSC) curve showing an overall resolution is 3.07 Å at FSC=0.143. **(e)** Angular distribution of the particles used in the final reconstruction. **(f)** Density map according to local resolution estimation. **(g)** Cryo-EM density

maps and models of the seven transmembrane helices (TM1-7), α5 helix of miniGq and the ligand of Butyrate bound FFA2-miniGq complex are shown. The EM density is shown at 0.152 threshold

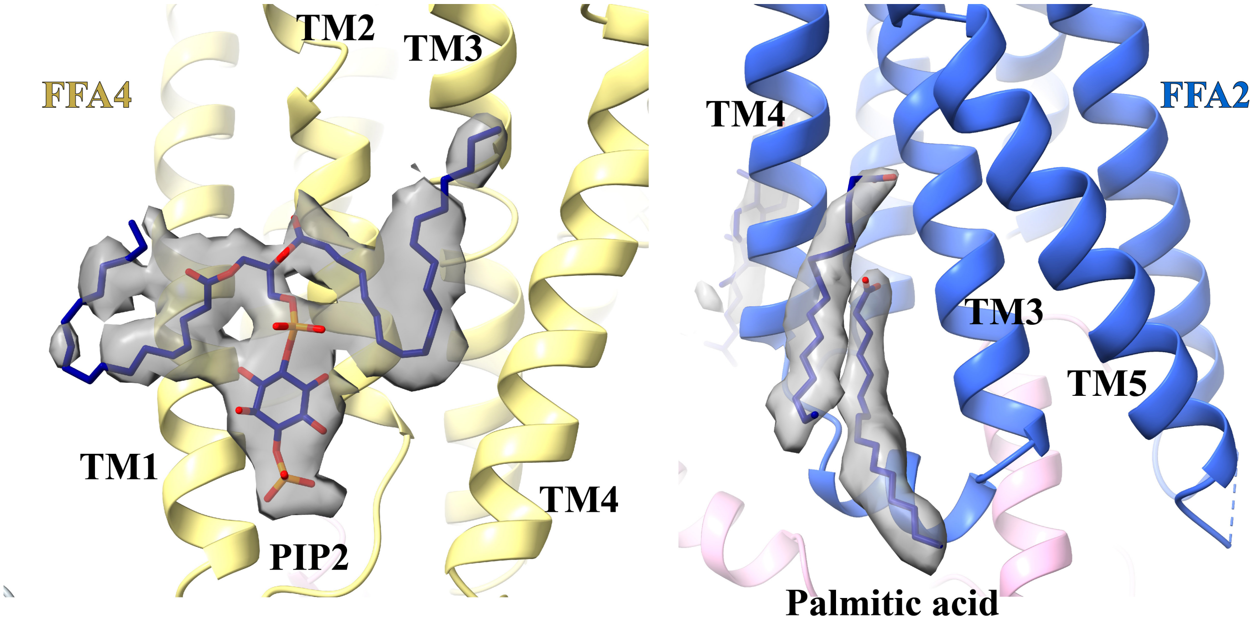

Figure S6. Lipid molecules surrounding 7TMs of FFA2 (blue) and FFA4 (yellow). PIP2 and palmitic acid molecules shown as dark blue sticks are modeled to fit the cryo-EM density (dark grey). The cryo-EM density of PIP2 is contoured at level 0.12. The cryo-EM density of palmitic acid is contoured at level 0.15.

A

Mutant	TUG-891 pEC$_{50}$	TUG-1197 pEC$_{50}$	DHA pEC$_{50}$
WT	7.03 ± 0.09	6.54 ± 0.13	4.83 ± 0.08
R22A	7.20 ± 0.04	6.70 ± 0.11	4.90 ± 0.02
R24A	7.19 ± 0.04	6.59 ± 0.12	4.83 ± 0.07
R22A/R24A	7.11 ± 0.07	6.58 ± 0.13	4.90 ± 0.04
D30A	6.91 ± 0.09	6.47 ± 0.08	4.73 ± 0.08
T119A	6.69 ± 0.04 (*)	5.69 ± 0.05 (***)	4.80 ± 0.14
E204A	6.69 ± 0.11 (*)	6.45 ± 0.10	4.82 ± 0.11
D208A	7.00 ± 0.08	6.58 ± 0.03	5.21 ± 0.09 (*)
I284A	6.66 ± 0.02 (*)	5.90 ± 0.15 (**)	5.05 ± 0.07
N291A	6.91 ± 0.10	6.56 ± 0.12	4.72 ± 0.09

Statistical significance : *, $p < 0.05$; **, $p < 0.01$; ***, $p < 0.001$

B

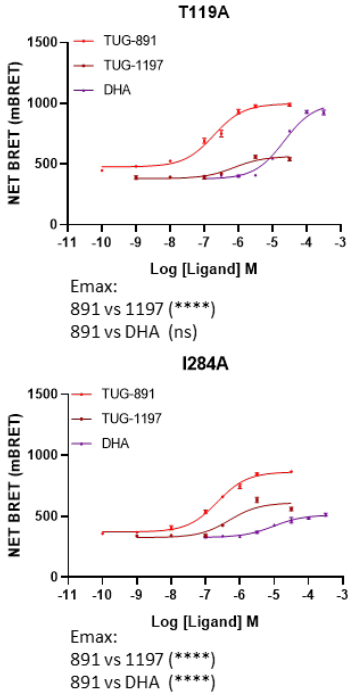

C

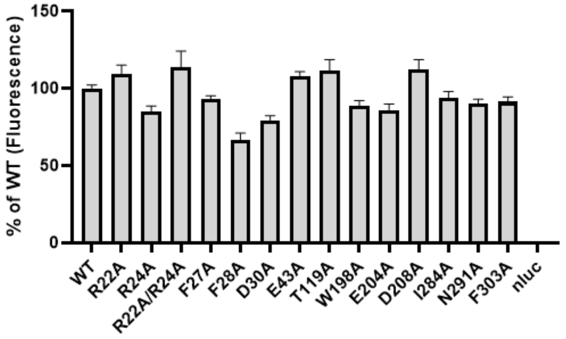

D

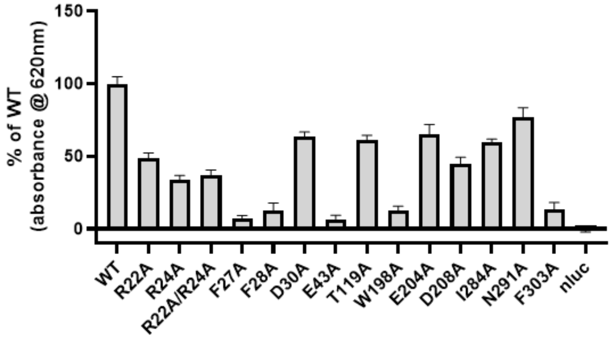

Figure S7. Mutagenesis studies on FFA4. (a) Point mutants of FFA4 were compared to wild type (WT) in arrestin-3 interaction studies to explore potential alterations in potency for TUG-891, TUG-1197 and DHA. Data are means ± S.E.M. for n = 3 or more. Significantly different from wild type at p < 0.05 *, < 0.01** and 0.001 ***. **(b)** Concentration-response curves for TUG-891, TUG-1197 and DHA at T119A (**upper panel**) and I284A (**lower panel**) highlight the reduced efficacy of DHA and TUG1197 at I284A but only for TUG-1197 at T119A. **(c)** whilst all FFA4-eYFP mutants were expressed effectively as measured by eYFP fluorescence, **(d)** only a subset was effectively delivered to the cell surface, as quantified by ELISA measurements. We were thus unable to define the effect of mutation to Ala of each of F27, F28 and E43 (see text for details).

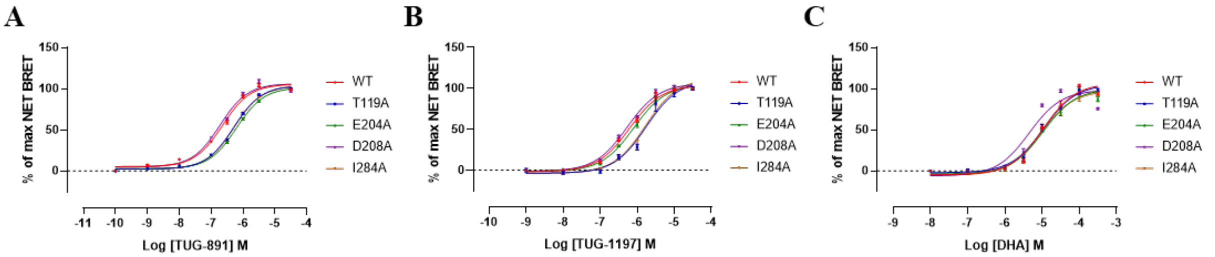

D

	TUG-891	TUG-1197	DHA
Mutant	pEC_{50}	pEC_{50}	pEC_{50}
WT	6.64 ± 0.04	6.12 ± 0.16	5.01 ± 0.04
T119A	6.29 ± 0.01 (****)	5.64 ± 0.20 (**)	4.97 ± 0.07
E204A	6.20 ± 0.04 (****)	6.07 ± 0.16	5.06 ± 0.05
D208A	6.73 ± 0.02	6.25 ± 0.17	5.36 ± 0.02 (**)
I284A	6.28 ± 0.02 (****)	5.79 ± 0.17 (**)	5.03 ± 0.09

Statistical significance : **, $p < 0.01$; ****, $p < 0.0001$

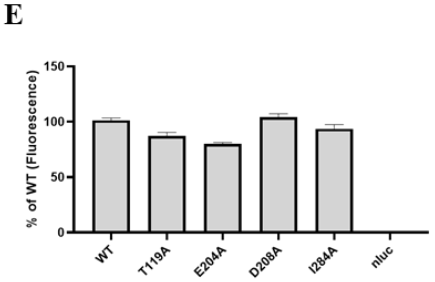

Figure S8. Contribution of key residues in FFA4 to agonist function studied with arrestin-2. Point mutants of key residues of FFA4 identified in arrestin-3 interaction studies (**Fig. S7**) were compared to wild type (WT) in arrestin-2 interaction studies to explore potential alterations in potency for (**a**) TUG-891, (**b**) TUG-1197 and (**c**) DHA. A summary of the outcomes with statistical significance is shown (**d**). Significantly different from wild type at p < 0.01** and 0.0001 ****. Data are means ± S.E.M. for n = 3 or more. Each of these FFA4-eYFP mutants was expressed as effectively as wild type as measured by eYFP fluorescence (**e**).

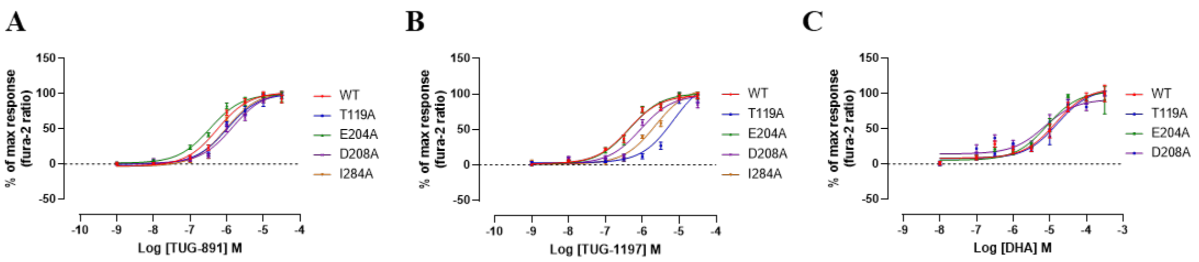

D

	TUG-891	TUG-1197	DHA
**Mutant**	**pEC_{50}**	**pEC_{50}**	**pEC_{50}**
WT	6.23 ± 0.06	6.36 ± 0.12	4.90 ± 0.37
T119A	5.98 ± 0.06 (**)	5.15 ± 0.10 (****)	4.89 ± 0.33
E204A	6.46 ± 0.01 (*)	6.34 ± 0.17	5.10 ± 0.22
D208A	5.83 ± 0.04 (***)	6.02 ± 0.09	5.14 ± 0.40
I284A	6.03 ± 0.03 (*)	5.65 ± 0.01 (**)	>4

Statistical significance : *, *p* < 0.05; **, *p* < 0.01; ***, *p* < 0.001; ****, *p* < 0.0001

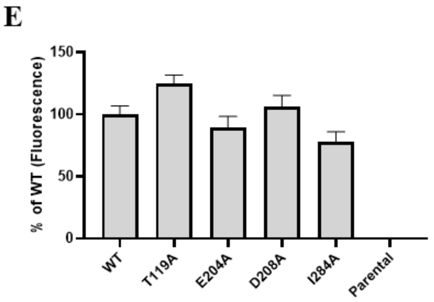

Figure S9. Contribution of key residues in FFA4 to agonist function assessed by Ca^{2+} mobilization assays. e-YFP-tagged forms of wild type (WT), T119A, E204A, D208A and I284A FFA4 were expressed stably in Flp-In^{TM} T-REx^{TM} 293 cells and their expression induced by overnight treatment with doxycycline (100 $ng.ml^{-1}$). Following loading of cells with Fura2-AM cells were treated with the indicated concentrations of TUG-891 (**a**), TUG-1197 (**b**) or DHA (**c**) and cytosolic Ca $^{2+}$ levels measured. A summary of the outcomes with statistical significance is shown (**d**). **NOTE**: Data for DHA at I284A FFA4 was not suitable for data fitting and is excluded and is simply recorded as > 4. Significantly different from wild type at $p < 0.05$*, $p < 0.01$**, $p < 0.001$*** and $p < 0.0001$ ****. Data are means ± S.E.M. for n = 3 or more. Levels of fluorescence corresponding to each form of FFA4-eYFP after doxycycline-induced construct expression is shown (**e**).

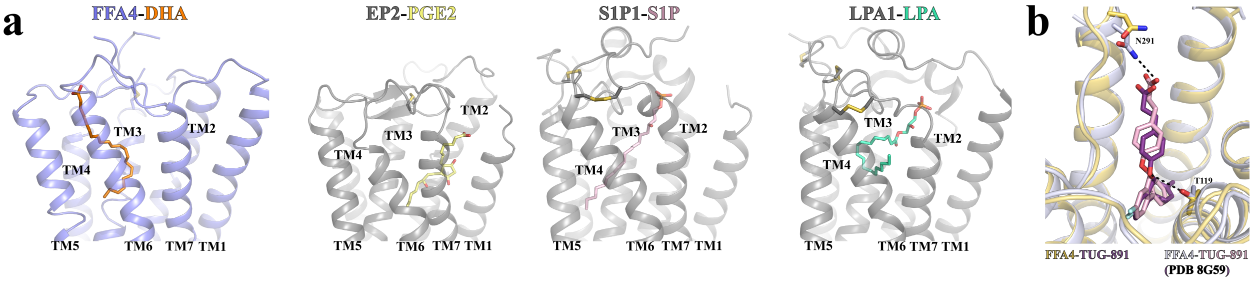

Figure S10. Structural comparison of FFA4 with (a) other lipid GPCRs and (b) published structure of FFA4 with TUG-891. EP2 is the receptor for the prostaglandin E2 (PGE2). S1P1 and LPA1 are receptors for the lysophospholipids S1P and LPA, respectively. The PDB IDs of the structures of EP2-PGE2, S1P1-S1P, and LPA1-LPA are 7XC2, 7TD3, and 7TD0, respectively. TUG-891 in our structure and in the published structure (PDB ID 8G59) is colored purple and pink, respectively.

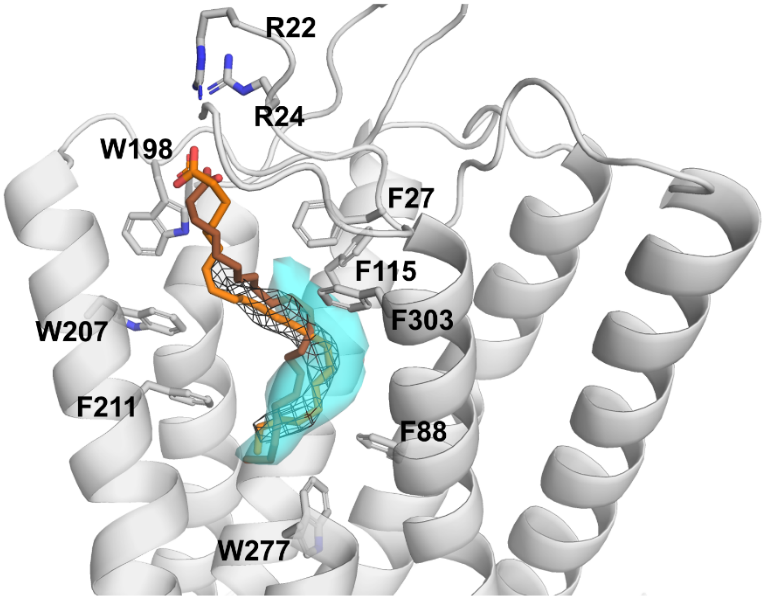

Figure S11. Comparison between the DHA binding mode to FFA4 observed in the cryo-EM structure (orange) and the mode most frequently observed in the MD simulations (brown). The cyan surface represents the ligand density calculated using the VMD VolMap tool at 1Å resolution on MD simulation trajectories (iso-value 0.1 g/mol*Å^3). The black mesh represents the cryo-EM density of the ligand (iso-value 5). The representative frame of the MD simulation was selected as the centroid of the most-populated cluster of conformations, identified using MDAnalysis.

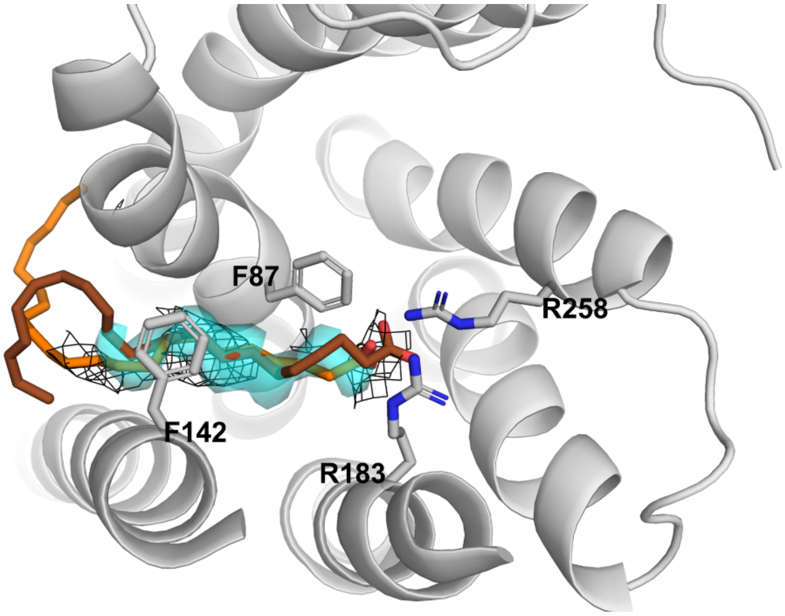

Figure S12. Comparison between the DHA binding mode to FFA1 observed in the cryo-EM structure (orange) and the mode most frequently seen in the MD simulations (brown). The cyan surface represents the ligand density calculated using the VMD VolMap tool at 1Å resolution on MD simulation trajectories (iso-value 0.1 g/mol*$Å^3$). The black mesh illustrates the cryo-EM density of the ligand (iso-value 2). The representative frame of MD simulation was selected as the centroid of the most-populated cluster of conformations identified using MDAnalysis.

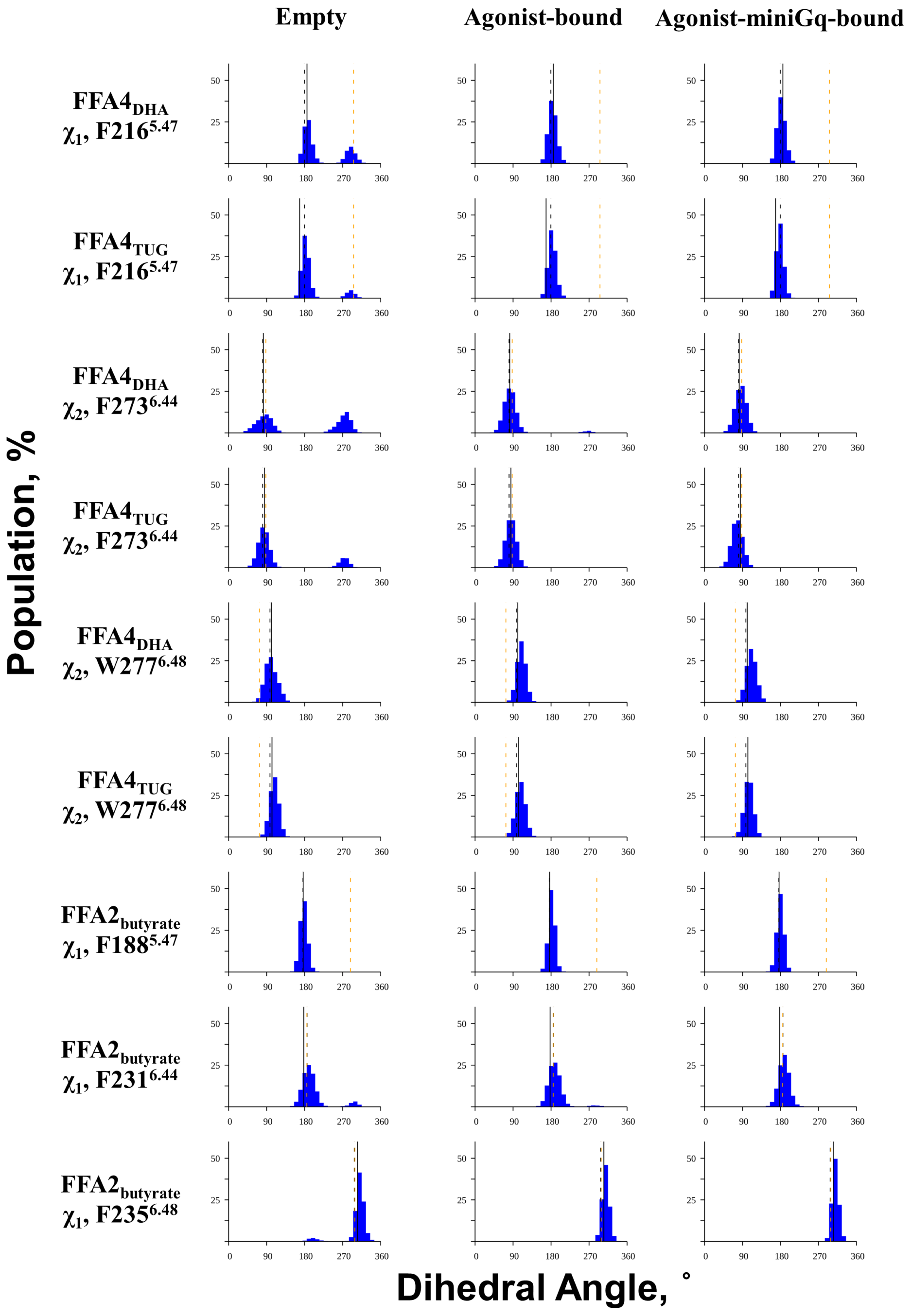

Figure S13. Dihedral angle population in MD simulations. The dihedral χ_1 or χ_2 angles of the aromatic residues in positions 5.47, 6.44, 6.45 and 6.48 are shown from the three 1 μs MD simulations of FFA4 and FFA2 in the empty, agonist- and agonist-miniGq-bound forms. The populations are calculated with bin width of 10°. The solid black lines show χ_1 / χ_2 observed in the cryoEM structure while the dashed black and orange lines represent AlphaFold predictions for the active and inactive state of the receptor, respectively.

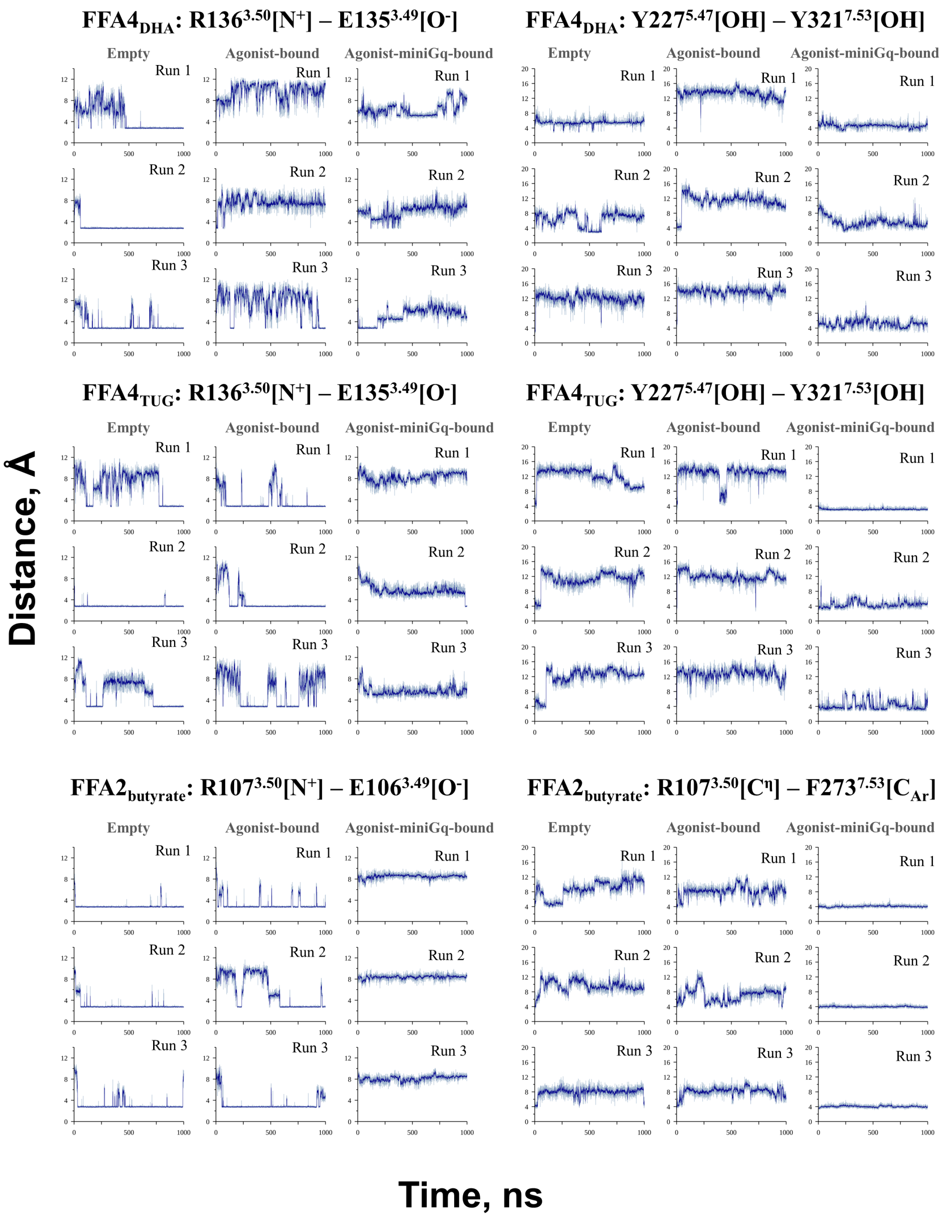

Figure S14. Evolution of atom distances in MD simulations. The distance between atoms of conserved amino acids known to be important for GPCR activation are shown from the three 1 µs MD simulations of FFA4 and FFA2 in the empty, agonist- and agonist-miniG$_q$-bound forms. The sets of atoms used to measure the minimum inter-atomic distance in each frame are given in brackets. The thin line represents the raw values while bold line represents the shifting average of 10 consecutive frames.

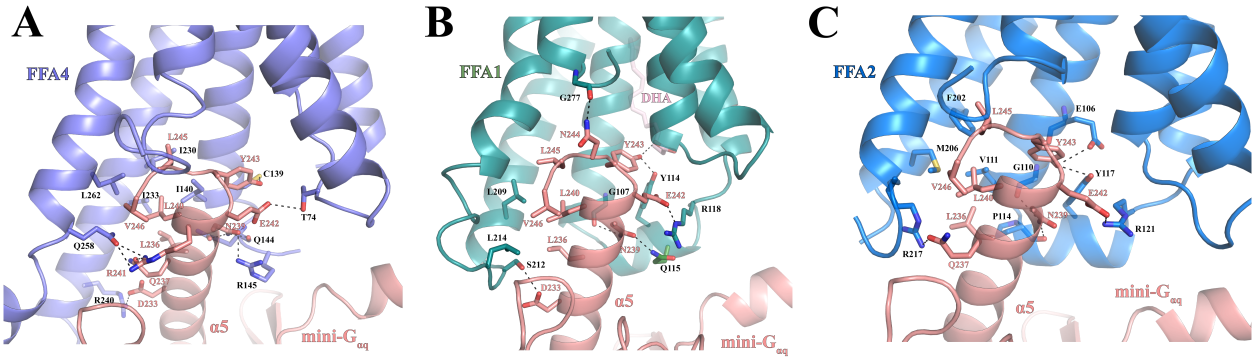

Figure S15. Interactions between FFAs and the α5 helix of mini-$G_{\alpha q}$. **(a)** Interactions between FFA4 and the α5 helix of mini-$G_{\alpha q}$. Specifically, hydrophobic interactions form among the mini-$G_{\alpha q}$ residues L236, L240, L245, and V246 and the FFA4 residues I140$^{3.54}$, I230$^{5.61}$, I233$^{5.64}$, and L262$^{6.33}$. In addition, Y243, E242, and D233 of mini-$G_{\alpha q}$ form polar interactions with the side chains of FFA4 residues C139$^{3.53}$, T74$^{2.39}$, and R240$^{5.71}$, respectively. Furthermore, N239 of mini-$G_{\alpha q}$ forms polar interactions with FFA4 residues R145^{ICL2} and Q144 ^{ICL2}, whereas R241 and Q237 of mini-$G_{\alpha q}$ form a polar interaction network with FFA4 residue Q258 $^{6.29}$. **(b)** Interactions between FFA1 and the α5 helix of mini-$G_{\alpha q}$. Specifically, hydrophobic interactions form among the mini-$G_{\alpha q}$ residues L236, L240, L245, and V246 and the FFA1 residues L209$^{5.65}$ and L214^{ICL3}. Y243, E242, N239, and D233 of mini-$G_{\alpha q}$ form polar interactions with the side chains of FFA1 residues Y114^{ICL2}, R118^{ICL2}, Q115^{ICL2}, and S212^{ICL3}, respectively. Furthermore, N244 and N239 of mini-$G_{\alpha q}$ forms polar interactions with the carbonyl groups of FFA1 residues G277$^{7.54}$ and G107$^{3.53}$, respectively. Y243 of mini-$G_{\alpha q}$ also forms a hydrogen bond with DHA that may be bound at 'Site 2'. **(c)** Interactions between FFA2 and the α5 helix of mini-$G_{\alpha q}$. Specifically, hydrophobic interactions form among the mini-$G_{\alpha q}$ residues L236, L240, L245, and V246 and the FFA2 residues M206$^{5.65}$ and F202$^{5.61}$. In addition, E242 and Q237 of mini-$G_{\alpha q}$ form polar interactions with the side chains of FFA2 residues R121^{ICL2} and R217$^{6.30}$, respectively. Furthermore, Y243 of mini-$G_{\alpha q}$ forms polar interactions with FFA2 residues E106$^{3.49}$ and Y117^{ICL2}, whereas 239 of mini-$G_{\alpha q}$ form polar interactions with the mainchain carbonyl groups of FFA4 residue G110$^{3.53}$ and P114 ^{ICL2}.

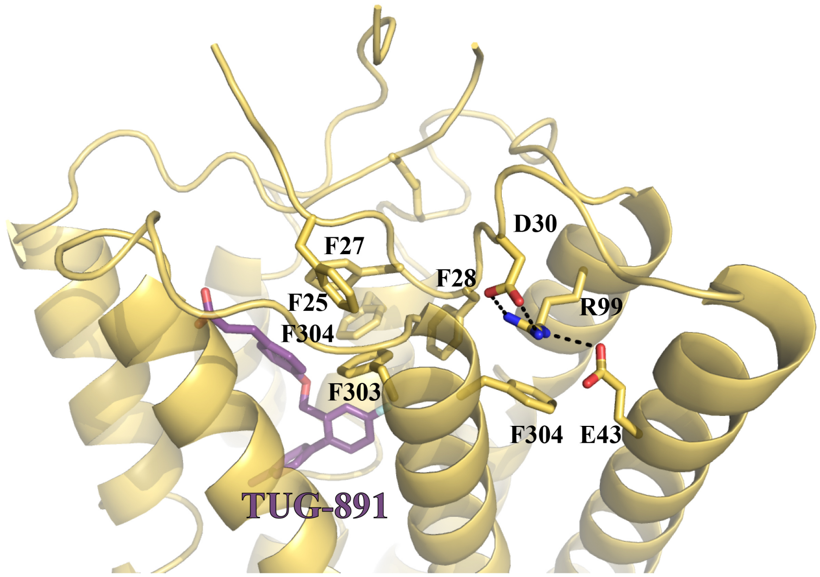

Figure S16. Interactions mediated by R99 of FFA4 and nearby aromatic network. In contrast to initial predictions based on homology modeling and mutagenesis, $R99^{2.64}$ does not interact directly with the carboxylate of TUG-891. Rather, it acts to shape the binding pocket through interactions with $D30^{N}$ and $E43^{1.35}$ and a cation-π interaction with $F304^{7.36}$.

Table S1. Cryo-EM data collection and refinement statistics 1

	(EMD-41007, PDB 8T3O)	(EMD-41008, PDB 8T3Q)
Data collection and processing	TUG-891-FFA4-miniGq	DHA-FFA4-miniGq
Magnification	105,000	105,000
Voltage (kV)	300	300
Electron exposure (e^-/Å^2)	55	55
Defocus range (μm)	-1.0 to -1.8	-1.0 to -1.8
Pixel size (Å)	0.828	0.828
Symmetry imposed	C1	C1
Initial particle images (no.)	4,928,436	9,585,265
Final particle images (no.)	391,203	380,284
Map resolution (Å)	3.06	3.14
FSC threshold	0.143	0.143
Map resolution range (Å)	2.5-4.5	2.5-4.5
Refinement		
Model resolution (Å)	3.3	3.4
FSC threshold	0.5	0.5
Model composition		
Non-hydrogen atoms	9112	9032
Protein residues	1158	1158
Ligand	1	1
R.m.s. deviations		
Bond lengths (Å)	0.005	0.008
Bond angles (°)	0.802	1.160
Validation		
MolProbity score	1.65	2.00
Clashscore	8.65	11.84
Rotamer outliers (%)	0.21	1.44
Ramachandran plot		
Favored (%)	96.93	95.79
Allowed (%)	3.07	4.21
Disallowed (%)	0	0

Table S2. Cryo-EM data collection and refinement statistics 2

	(EMD-41013, PDB 8T3V)/(EMD-41014)	(EMD-41010, PDB 8T3S)
Data collection and processing	DHA-FFA1-miniGq/Local	butyrate-FFA2-miniGq
Magnification	105,000	105,000
Voltage (kV)	300	300
Electron exposure (e^{-}/Å^{2})	61.6	61.6
Defocus range (μm)	-0.8 to -2.5	-0.8 to -2.5
Pixel size (Å)	0.826	0.826
Symmetry imposed	C1	C1
Initial particle images (no.)	7,942,319	12,559,721
Final particle images (no.)	305,318	393,952
Map resolution (Å)	3.39/3.6	3.07
FSC threshold	0.143	0.143
Map resolution range (Å)	2.5-4.5	2.5-4.5
Refinement		
Model resolution (Å)	4.0/3.8	3.5
FSC threshold	0.5	0.5
Model composition		
Non-hydrogen atoms	8745	8772
Protein residues	1136	1119
Ligand	1	1
R.m.s. deviations		
Bond lengths (Å)	0.007	0.007
Bond angles (°)	0.873	0.797
Validation		
MolProbity score	1.69	1.60
Clashscore	11.48	9.17
Rotamer outliers (%)	0.44	0.53
Ramachandran plot		
Favored (%)	97.41	97.45
Allowed (%)	2.59	2.55
Disallowed (%)	0	0

Table S3. The average root mean square deviation and fluctuation (RMSD and RMSF) values observed for the simulated systems. RMSD and RMSF values were calculated for the receptor Cα atoms, the 7- transmembrane bundle (7TMB) Cα atoms and the ligand non-hydrogen atoms (Ligand) from the three 1μ MD simulations. RMSD values were averaged within each replica and combined between different replicas to yield the mean ± standard deviation values given below. RMSF values are also given as mean ± standard deviation between all replicas.

Receptor Systems	RMSD, Å			RMSF, Å		
	Cα	7TMB-Cα	Ligand	Cα	7TMB-Cα	Ligand
$FFA4_{DHA}$	3.6 ± 0.2	1.3 ± 0.0	4.5 ± 1.1	1.3 ± 0.1	0.7 ± 0.0	1.9 ± 0.2
$FFA4_{empty}$	3.7 ± 0.1	1.5 ± 0.0		1.5 ± 0.2	0.8 ± 0.1	
$FFA4_{DHA\_Gq}$	3.5 ± 0.1	1.4 ± 0.1	4.6 ± 0.5	1.3 ± 0.1	0.7 ± 0.0	2.3 ± 0.1
$FFA4_{TUG\text{-}891}$	3.5 ± 0.3	1.2 ± 0.0	1.7 ± 0.2	1.3 ± 0.1	0.7 ± 0.1	1.0 ± 0.1
$FFA4_{empty\_TUG\text{-}891}$	3.2 ± 0.4	1.3 ± 0.1		1.4 ± 0.2	0.8 ± 0.1	
$FFA4_{TUG\text{-}891\_Gq}$	2.7 ± 0.1	1.1 ± 0.1	2.3 ± 0.9	1.1 ± 0.1	0.6 ± 0.0	1.2 ± 0.3
$FFA2_{butyrate}$	2.6 ± 0.2	1.1 ± 0.0	2.0 ± 0.4	1.1 ± 0.0	0.7 ± 0.0	1.6 ± 0.2
$FFA2_{empty}$	2.8 ± 0.1	1.3 ± 0.2		1.2 ± 0.1	0.8 ± 0.1	
$FFA2_{butyrate\_Gq}$	2.4 ± 0.1	1.1 ± 0.1	2.4 ± 0.7	1.0 ± 0.1	0.7 ± 0.1	1.4 ± 0.1

Table S4. The average ligand fragment-residue interaction energy. The interaction energy involving electrostatic (ele) and van der Waals (vdW) components in kcal/mol was calculated from three 1μ MD simulations of the FFA4-agonist complexes. The fragmentation of the agonists is shown below. The residues having noticeable interactions with a given ligand fragment, i.e. having total energy below -1 kcal/mol or above 1 kcal/mol are shown in bold. Among them, residues having repulsive interactions, i.e. positive total energy, are colored red.
The values were averaged within each replica and combined between different replicas to yield the mean ± standard deviation values given below.

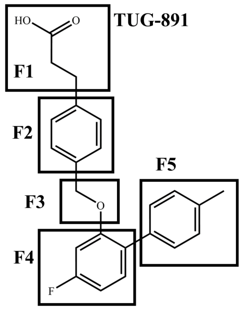

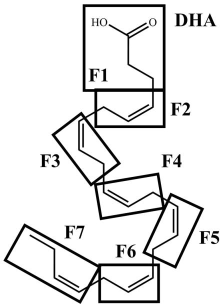

TUG-891, F1				TUG-891, F2			
	ele	vdW	total		ele	vdW	total
**R22**	**-59 ± 10**	**1.8 ± 0.5**	**-58 ± 10**	**I284**	**-0.3 ± 0.0**	**-2.0 ± 0.0**	**-2.3 ± 0.1**
**R24**	**-22 ± 14**	**-0.3 ± 0.2**	**-23 ± 14**	**W207**	**-0.3 ± 0.0**	**-0.8 ± 0.1**	**-1.1 ± 0.1**
**W198**	**-12 ± 2**	**-0.3 ± 0.1**	**-12 ± 2**	**F115**	**-0.3 ± 0.1**	**-0.7 ± 0.3**	**-1.0 ± 0.4**
**D208**	**-2.9 ± 0.8**	**-0.3 ± 0.0**	**-3.2 ± 0.7**	F303	-0.2 ± 0.1	-0.7 ± 0.1	-0.9 ± 0.2
**I287**	**-1.3 ± 0.2**	**-1.4 ± 0.1**	**-2.7 ± 0.1**	F27	-0.2 ± 0.1	-0.6 ± 0.3	-0.8 ± 0.3
**F27**	**-1.8 ± 0.1**	**-0.7 ± 0.0**	**-2.4 ± 0.0**	I280	0.2 ± 0.0	-0.7 ± 0.1	-0.6 ± 0.1
**N291**	**-1.9 ± 0.5**	**-0.0 ± 0.0**	**-1.9 ± 0.5**	L173	0.2 ± 0.0	-0.7 ± 0.1	-0.5 ± 0.0
**F25**	**-0.7 ± 1.9**	**-0.8 ± 0.1**	**-1.6 ± 2.0**	F211	-0.2 ± 0.0	-0.3 ± 0.1	-0.5 ± 0.1
W207	-0.6 ± 0.1	-0.3 ± 0.1	-0.9 ± 0.2	I287	0.1 ± 0.0	-0.5 ± 0.0	-0.4 ± 0.1
L173	-0.3 ± 0.1	-0.2 ± 0.1	-0.5 ± 0.1	T283	-0.0 ± 0.0	-0.4 ± 0.0	-0.4 ± 0.1
F115	-0.2 ± 0.1	-0.1 ± 0.1	-0.4 ± 0.2	L196	-0.1 ± 0.0	-0.3 ± 0.1	-0.4 ± 0.2
F303	-0.1 ± 0.1	-0.2 ± 0.1	-0.3 ± 0.2	T119	0.0 ± 0.0	-0.4 ± 0.1	-0.4 ± 0.1
I284	0.1 ± 0.2	-0.5 ± 0.0	-0.3 ± 0.2	F25	-0.1 ± 0.0	-0.2 ± 0.1	-0.3 ± 0.1
I280	-0.1 ± 0.0	-0.0 ± 0.0	-0.1 ± 0.0	W198	-0.1 ± 0.1	-0.1 ± 0.0	-0.2 ± 0.1
I197	0.1 ± 0.1	-0.0 ± 0.0	0.1 ± 0.1	D208	-0.0 ± 0.0	-0.2 ± 0.0	-0.2 ± 0.0
T283	0.3 ± 0.1	-0.1 ± 0.0	0.2 ± 0.1	I281	-0.1 ± 0.0	-0.0 ± 0.0	-0.2 ± 0.0
L288	0.3 ± 0.1	-0.0 ± 0.0	0.2 ± 0.1	E204	-0.1 ± 0.0	-0.0 ± 0.0	-0.1 ± 0.0
L196	1.8 ± 1.3	-1.4 ± 0.2	0.5 ± 1.1	R22	-0.1 ± 0.0	-0.0 ± 0.0	-0.1 ± 0.0
**Q290**	**2.2 ± 0.4**	**-0.0 ± 0.0**	**2.2 ± 0.3**	T310	-0.0 ± 0.0	-0.0 ± 0.0	-0.0 ± 0.0
**E204**	**27 ± 0**	**-0.4 ± 0.0**	**26 ± 0**	M118	0.2 ± 0.0	-0.0 ± 0.0	0.1 ± 0.0
**TUG-891, F3**				**TUG-891, F4**			
	ele	vdW	total		ele	vdW	total
**F115**	**-1.1 ± 0.2**	**-0.5 ± 0.2**	**-1.6 ± 0.4**	**M118**	**-0.8 ± 0.1**	**-2.1 ± 0.2**	**-2.9 ± 0.1**
**T119**	**-0.9 ± 0.1**	**-0.5 ± 0.1**	**-1.4 ± 0.1**	**I280**	**-0.2 ± 0.0**	**-1.6 ± 0.0**	**-1.8 ± 0.1**
T283	-0.8 ± 0.0	-0.0 ± 0.0	-0.9 ± 0.0	**T119**	**0.4 ± 0.0**	**-1.8 ± 0.5**	**-1.4 ± 0.5**

I284	-0.3 ± 0.0	-0.3 ± 0.0	-0.7 ± 0.0	**F88**	**-0.3 ± 0.1**	**-0.8 ± 0.0**	**-1.2 ± 0.1**
F27	-0.3 ± 0.0	-0.1 ± 0.0	-0.4 ± 0.1	**V307**	**-0.3 ± 0.2**	**-0.9 ± 0.2**	**-1.1 ± 0.4**
F211	-0.1 ± 0.0	-0.2 ± 0.1	-0.3 ± 0.0	T310	-0.1 ± 0.3	-0.8 ± 0.2	-0.9 ± 0.5
F303	-0.2 ± 0.1	-0.1 ± 0.0	-0.3 ± 0.1	W277	-0.4 ± 0.0	-0.4 ± 0.0	-0.8 ± 0.1
L120	-0.2 ± 0.0	-0.0 ± 0.0	-0.2 ± 0.0	G122	-0.3 ± 0.1	-0.4 ± 0.2	-0.7 ± 0.3
W207	-0.1 ± 0.0	-0.1 ± 0.0	-0.2 ± 0.0	F115	0.3 ± 0.1	-0.8 ± 0.0	-0.5 ± 0.1
F25	-0.1 ± 0.0	-0.0 ± 0.0	-0.1 ± 0.0	F303	-0.1 ± 0.0	-0.3 ± 0.2	-0.4 ± 0.2
I280	0.4 ± 0.1	-0.5 ± 0.0	-0.1 ± 0.1	F311	-0.0 ± 0.1	-0.4 ± 0.1	-0.4 ± 0.2
V307	-0.1 ± 0.0	-0.0 ± 0.0	-0.1 ± 0.0	F211	-0.2 ± 0.0	-0.1 ± 0.0	-0.4 ± 0.1
L173	0.0 ± 0.0	-0.1 ± 0.0	-0.1 ± 0.0	F28	-0.2 ± 0.1	-0.1 ± 0.1	-0.3 ± 0.2
F88	0.1 ± 0.0	-0.0 ± 0.0	0.1 ± 0.0	S123	-0.2 ± 0.0	-0.1 ± 0.1	-0.3 ± 0.1
T310	0.1 ± 0.0	-0.0 ± 0.0	0.1 ± 0.0	F27	-0.1 ± 0.1	-0.1 ± 0.1	-0.2 ± 0.2
M118	0.3 ± 0.1	-0.1 ± 0.0	0.2 ± 0.1	I284	-0.0 ± 0.0	-0.1 ± 0.0	-0.1 ± 0.0
S123	0.2 ± 0.0	-0.0 ± 0.0	0.2 ± 0.0	W207	-0.1 ± 0.0	-0.0 ± 0.0	-0.1 ± 0.0
I281	0.3 ± 0.0	-0.0 ± 0.0	0.3 ± 0.0	L173	-0.0 ± 0.0	-0.0 ± 0.0	-0.1 ± 0.0
G122	0.5 ± 0.1	-0.0 ± 0.0	0.5 ± 0.1	N215	-0.0 ± 0.0	-0.0 ± 0.0	-0.0 ± 0.0
	**TUG-891, F5**				**DHA, F1**		
	ele	vdW	total		ele	vdW	total
**F211**	**-0.7 ± 0.1**	**-2.2 ± 0.4**	**-3.0 ± 0.5**	**R24**	**-52 ± 27**	**0.0 ± 0.9**	**-52 ± 26**
**T119**	**-0.3 ± 0.1**	**-1.6 ± 0.2**	**-1.9 ± 0.1**	**R22**	**-27 ± 21**	**0.2 ± 0.3**	**-27 ± 20**
**I280**	**-0.4 ± 0.0**	**-1.3 ± 0.1**	**-1.7 ± 0.1**	**W198**	**-2.3 ± 1.1**	**-0.1 ± 0.1**	**-2.4 ± 1.1**
**W277**	**-0.1 ± 0.1**	**-1.3 ± 0.3**	**-1.4 ± 0.4**	**F27**	**-1.0 ± 0.8**	**-0.6 ± 0.2**	**-1.6 ± 1.0**
**I281**	**-0.1 ± 0.0**	**-1.0 ± 0.1**	**-1.1 ± 0.1**	F25	-0.0 ± 3.5	-0.8 ± 0.5	-0.8 ± 3.8
**S123**	**0.1 ± 0.1**	**-1.1 ± 0.1**	**-1.0 ± 0.0**	I287	-0.1 ± 0.4	-0.4 ± 0.3	-0.5 ± 0.4
M118	-0.5 ± 0.3	-0.4 ± 0.2	-0.8 ± 0.4	L173	-0.1 ± 0.2	-0.2 ± 0.2	-0.3 ± 0.4
N215	0.3 ± 0.1	-1.0 ± 0.2	-0.7 ± 0.1	F303	-0.2 ± 0.2	-0.1 ± 0.1	-0.3 ± 0.3
F216	0.0 ± 0.1	-0.7 ± 0.1	-0.7 ± 0.1	F115	0.0 ± 0.1	-0.2 ± 0.2	-0.2 ± 0.1
V212	-0.1 ± 0.0	-0.5 ± 0.2	-0.6 ± 0.2	W207	-0.1 ± 0.1	-0.0 ± 0.0	-0.1 ± 0.1
I126	0.4 ± 0.0	-0.9 ± 0.1	-0.5 ± 0.1	I284	-0.0 ± 0.1	-0.1 ± 0.0	-0.1 ± 0.1
G122	0.3 ± 0.1	-0.8 ± 0.0	-0.5 ± 0.1	F28	-0.0 ± 0.0	-0.0 ± 0.0	-0.0 ± 0.0
W207	-0.2 ± 0.0	-0.1 ± 0.0	-0.3 ± 0.0	I201	-0.0 ± 0.0	-0.0 ± 0.0	-0.0 ± 0.0
I284	0.3 ± 0.0	-0.7 ± 0.0	-0.3 ± 0.0	I280	-0.0 ± 0.0	-0.0 ± 0.0	-0.0 ± 0.0
F88	-0.1 ± 0.0	-0.2 ± 0.1	-0.3 ± 0.2	L288	0.1 ± 0.1	-0.0 ± 0.0	0.1 ± 0.1
L120	-0.1 ± 0.1	-0.1 ± 0.0	-0.3 ± 0.0	L196	1.8 ± 3.2	-1.5 ± 0.1	0.3 ± 3.1
D208	-0.2 ± 0.1	-0.0 ± 0.0	-0.2 ± 0.1	Q290	0.4 ± 0.4	-0.0 ± 0.0	0.4 ± 0.4
T310	0.0 ± 0.0	-0.1 ± 0.0	-0.1 ± 0.0	N291	0.4 ± 0.6	-0.0 ± 0.0	0.4 ± 0.6
L285	0.1 ± 0.0	-0.0 ± 0.0	0.1 ± 0.0	I197	0.6 ± 0.4	-0.1 ± 0.0	0.5 ± 0.5
V124	0.1 ± 0.0	-0.0 ± 0.0	0.1 ± 0.0	**D208**	**2.9 ± 2.1**	**-0.0 ± 0.0**	**2.9 ± 2.1**
T283	0.5 ± 0.0	-0.0 ± 0.0	0.5 ± 0.0	**E204**	**10 ± 6**	**-0.1 ± 0.1**	**10 ± 6**
	**DHA, F2**				**DHA, F3**		
	ele	vdW	total		ele	vdW	total
**R24**	**-0.8 ± 0.1**	**-0.2 ± 0.3**	**-1.1 ± 0.4**	F115	-0.0 ± 0.1	-0.8 ± 0.3	-0.8 ± 0.2
F115	-0.0 ± 0.1	-0.7 ± 0.5	-0.8 ± 0.5	F27	-0.0 ± 0.0	-0.5 ± 0.2	-0.5 ± 0.2

F27	-0.1 ± 0.1	-0.6 ± 0.2	-0.7 ± 0.2	F303	-0.1 ± 0.1	-0.4 ± 0.2	-0.4 ± 0.3
R22	-0.6 ± 0.5	-0.1 ± 0.0	-0.6 ± 0.5	F25	0.0 ± 0.1	-0.4 ± 0.5	-0.4 ± 0.5
L196	-0.1 ± 0.0	-0.4 ± 0.1	-0.5 ± 0.1	L173	0.0 ± 0.0	-0.3 ± 0.1	-0.3 ± 0.0
F25	0.2 ± 0.3	-0.6 ± 0.4	-0.4 ± 0.3	M118	-0.0 ± 0.1	-0.3 ± 0.3	-0.3 ± 0.4
I287	-0.1 ± 0.1	-0.4 ± 0.4	-0.4 ± 0.5	I284	0.0 ± 0.0	-0.3 ± 0.2	-0.3 ± 0.2
L173	-0.0 ± 0.0	-0.4 ± 0.3	-0.4 ± 0.3	L196	-0.0 ± 0.0	-0.2 ± 0.2	-0.3 ± 0.2
F303	-0.1 ± 0.1	-0.3 ± 0.2	-0.3 ± 0.2	I280	-0.0 ± 0.0	-0.2 ± 0.1	-0.2 ± 0.1
I284	0.0 ± 0.0	-0.2 ± 0.2	-0.2 ± 0.2	W207	-0.1 ± 0.0	-0.1 ± 0.1	-0.2 ± 0.1
I280	-0.1 ± 0.1	-0.0 ± 0.0	-0.1 ± 0.1	R24	0.0 ± 0.1	-0.2 ± 0.3	-0.2 ± 0.3
T119	0.0 ± 0.0	-0.1 ± 0.1	-0.1 ± 0.1	T119	0.1 ± 0.1	-0.2 ± 0.1	-0.1 ± 0.0
W207	-0.0 ± 0.0	-0.1 ± 0.1	-0.1 ± 0.1	V307	0.0 ± 0.0	-0.2 ± 0.1	-0.1 ± 0.1
W198	-0.1 ± 0.1	-0.0 ± 0.0	-0.1 ± 0.1	D208	-0.1 ± 0.1	-0.0 ± 0.0	-0.1 ± 0.1
F28	-0.0 ± 0.0	-0.0 ± 0.1	-0.1 ± 0.1	I287	-0.0 ± 0.0	-0.1 ± 0.1	-0.1 ± 0.1
E204	0.1 ± 0.2	-0.0 ± 0.0	0.1 ± 0.2	F28	-0.0 ± 0.0	-0.1 ± 0.1	-0.1 ± 0.1
		**DHA, F4**				**DHA, F5**	
	ele	vdW	total		ele	vdW	total
F115	-0.1 ± 0.0	-0.8 ± 0.2	-0.9 ± 0.3	**M118**	**-0.3 ± 0.1**	**-1.1 ± 0.2**	**-1.4 ± 0.2**
M118	-0.1 ± 0.2	-0.8 ± 0.2	-0.9 ± 0.5	T119	0.0 ± 0.1	-0.8 ± 0.1	-0.7 ± 0.1
V307	-0.1 ± 0.1	-0.5 ± 0.3	-0.5 ± 0.4	I280	0.1 ± 0.0	-0.7 ± 0.1	-0.6 ± 0.1
T119	0.1 ± 0.0	-0.4 ± 0.1	-0.4 ± 0.1	F88	-0.1 ± 0.0	-0.4 ± 0.2	-0.5 ± 0.2
I280	0.0 ± 0.0	-0.4 ± 0.1	-0.3 ± 0.1	F115	-0.1 ± 0.0	-0.3 ± 0.2	-0.4 ± 0.2
F303	-0.0 ± 0.1	-0.3 ± 0.2	-0.3 ± 0.2	V307	-0.0 ± 0.0	-0.4 ± 0.0	-0.4 ± 0.0
F25	0.0 ± 0.1	-0.3 ± 0.4	-0.3 ± 0.4	T310	-0.0 ± 0.1	-0.3 ± 0.2	-0.3 ± 0.3
F27	-0.0 ± 0.0	-0.2 ± 0.2	-0.3 ± 0.1	W277	-0.1 ± 0.1	-0.2 ± 0.1	-0.3 ± 0.2
L173	-0.0 ± 0.0	-0.2 ± 0.1	-0.2 ± 0.1	F303	-0.2 ± 0.1	-0.1 ± 0.1	-0.3 ± 0.2
F311	-0.0 ± 0.0	-0.2 ± 0.2	-0.2 ± 0.2	F211	0.0 ± 0.0	-0.1 ± 0.0	-0.1 ± 0.0
F88	-0.0 ± 0.1	-0.1 ± 0.1	-0.2 ± 0.2	I284	0.0 ± 0.0	-0.1 ± 0.1	-0.1 ± 0.1
I284	0.0 ± 0.0	-0.2 ± 0.1	-0.2 ± 0.1	F25	0.0 ± 0.0	-0.1 ± 0.2	-0.1 ± 0.2
R24	-0.1 ± 0.1	-0.0 ± 0.1	-0.1 ± 0.1	F27	-0.0 ± 0.0	-0.1 ± 0.1	-0.1 ± 0.1
W207	-0.0 ± 0.0	-0.1 ± 0.1	-0.1 ± 0.1	F311	0.0 ± 0.1	-0.1 ± 0.1	-0.1 ± 0.0
F28	-0.0 ± 0.0	-0.1 ± 0.0	-0.1 ± 0.1	L173	0.0 ± 0.0	-0.1 ± 0.0	-0.1 ± 0.0
T310	0.1 ± 0.1	-0.1 ± 0.1	-0.1 ± 0.1	W207	-0.0 ± 0.0	-0.1 ± 0.0	-0.1 ± 0.1
F211	-0.0 ± 0.0	-0.1 ± 0.0	-0.1 ± 0.0	I281	-0.0 ± 0.0	-0.0 ± 0.0	-0.1 ± 0.0
L196	-0.0 ± 0.0	-0.0 ± 0.0	-0.1 ± 0.0	T283	0.1 ± 0.0	-0.0 ± 0.0	0.1 ± 0.0
G122	0.1 ± 0.0	-0.0 ± 0.0	0.1 ± 0.0	G122	0.2 ± 0.1	-0.1 ± 0.1	0.1 ± 0.1
		**DHA, F6**				**DHA, F7**	
	ele	vdW	total		ele	vdW	total
**T119**	**-0.1 ± 0.1**	**-1.1 ± 0.1**	**-1.2 ± 0.2**	**G122**	**-0.6 ± 0.1**	**-0.7 ± 0.1**	**-1.3 ± 0.1**
M118	-0.2 ± 0.2	-0.7 ± 0.1	-0.9 ± 0.3	**F211**	**-0.2 ± 0.0**	**-1.1 ± 0.1**	**-1.3 ± 0.1**
I280	-0.0 ± 0.0	-0.7 ± 0.1	-0.8 ± 0.1	**W277**	**-0.1 ± 0.1**	**-1.1 ± 0.1**	**-1.2 ± 0.1**
W277	-0.2 ± 0.0	-0.4 ± 0.0	-0.6 ± 0.1	**I280**	**-0.2 ± 0.0**	**-0.9 ± 0.1**	**-1.0 ± 0.0**
F211	-0.0 ± 0.0	-0.4 ± 0.1	-0.5 ± 0.1	S123	-0.1 ± 0.0	-0.7 ± 0.1	-0.7 ± 0.1
F88	-0.1 ± 0.0	-0.3 ± 0.1	-0.4 ± 0.1	N215	-0.0 ± 0.0	-0.7 ± 0.1	-0.7 ± 0.1

G122	-0.0 ± 0.2	-0.3 ± 0.0	-0.3 ± 0.2	I281	0.0 ± 0.0	-0.6 ± 0.1	-0.6 ± 0.1
I284	0.0 ± 0.0	-0.2 ± 0.0	-0.2 ± 0.0	I126	0.1 ± 0.0	-0.6 ± 0.1	-0.5 ± 0.1
V307	-0.1 ± 0.0	-0.1 ± 0.1	-0.2 ± 0.1	F216	-0.0 ± 0.0	-0.5 ± 0.2	-0.5 ± 0.2
T310	0.0 ± 0.0	-0.2 ± 0.1	-0.2 ± 0.0	I284	0.0 ± 0.0	-0.4 ± 0.1	-0.3 ± 0.1
W207	-0.1 ± 0.0	-0.1 ± 0.0	-0.1 ± 0.0	T119	0.4 ± 0.0	-0.7 ± 0.1	-0.3 ± 0.1
I281	0.0 ± 0.0	-0.1 ± 0.0	-0.1 ± 0.0	F88	-0.1 ± 0.0	-0.2 ± 0.1	-0.3 ± 0.1
S123	0.1 ± 0.0	-0.2 ± 0.0	-0.1 ± 0.1	W207	-0.1 ± 0.0	-0.1 ± 0.1	-0.2 ± 0.1
F303	-0.1 ± 0.0	-0.0 ± 0.0	-0.1 ± 0.1	V212	-0.0 ± 0.0	-0.2 ± 0.1	-0.2 ± 0.1
L120	-0.0 ± 0.0	-0.1 ± 0.0	-0.1 ± 0.0	T310	0.0 ± 0.0	-0.2 ± 0.1	-0.1 ± 0.1
F311	-0.0 ± 0.0	-0.0 ± 0.0	-0.1 ± 0.0	D208	-0.1 ± 0.0	-0.0 ± 0.0	-0.1 ± 0.1
D208	-0.1 ± 0.0	-0.0 ± 0.0	-0.1 ± 0.0	M118	0.2 ± 0.1	-0.3 ± 0.2	-0.1 ± 0.2
F25	0.0 ± 0.0	-0.1 ± 0.1	-0.0 ± 0.1	V307	-0.0 ± 0.0	-0.1 ± 0.1	-0.1 ± 0.1
T283	0.1 ± 0.0	-0.0 ± 0.0	0.1 ± 0.0	T283	0.1 ± 0.0	-0.0 ± 0.0	0.1 ± 0.0

REFERENCES AND NOTES

1. E. Alvarez-Curto, G. Milligan, Metabolism meets immunity: The role of free fatty acid receptors in the immune system. *Biochem. Pharmacol.* **114**, 3–13 (2016).

2. I. Kimura, A. Ichimura, R. Ohue-Kitano, M. Igarashi, Free fatty acid receptors in health and disease. *Physiol. Rev.* **100**, 171–210 (2020).

3. D. Y. Oh, S. Talukdar, E. J. Bae, T. Imamura, H. Morinaga, W. Fan, P. Li, W. J. Lu, S. M. Watkins, J. M. Olefsky, GPR120 is an omega-3 fatty acid receptor mediating potent anti-inflammatory and insulin-sensitizing effects. *Cell* **142**, 687–698 (2010).

4. C. P. Briscoe, M. Tadayyon, J. L. Andrews, W. G. Benson, J. K. Chambers, M. M. Eilert, C. Ellis, N. A. Elshourbagy, A. S. Goetz, D. T. Minnick, P. R. Murdock, H. R. Sauls, Jr., U. Shabon, L. D. Spinage, J. C. Strum, P. G. Szekeres, K. B. Tan, J. M. Way, D. M. Ignar, S. Wilson, A. I. Muir, The orphan G protein-coupled receptor GPR40 is activated by medium and long chain fatty acids. *J. Biol. Chem.* **278**, 11303–11311 (2003).

5. J. L. McCarville, G. Y. Chen, V. D. Cuevas, K. Troha, J. S. Ayres, Microbiota metabolites in health and disease. *Annu. Rev. Immunol.* **38**, 147–170 (2020).

6. Y. Itoh, Y. Kawamata, M. Harada, M. Kobayashi, R. Fujii, S. Fukusumi, K. Ogi, M. Hosoya, Y. Tanaka, H. Uejima, H. Tanaka, M. Maruyama, R. Satoh, S. Okubo, H. Kizawa, H. Komatsu, F. Matsumura, Y. Noguchi, T. Shinohara, S. Hinuma, Y. Fujisawa, M. Fujino, Free fatty acids regulate insulin secretion from pancreatic β cells through GPR40. *Nature* **422**, 173–176 (2003).

7. Z. Li, Z. Zhou, L. Zhang, Current status of GPR40/FFAR1 modulators in medicinal chemistry (2016-2019): A patent review. *Expert Opin. Ther. Pat.* **30**, 27–38 (2020).

8. A. D. Mancini, V. Poitout, The fatty acid receptor FFA1/GPR40 a decade later: How much do we know? *Trends Endocrinol. Metab.* **24**, 398–407 (2013).

9. A. D. Mancini, V. Poitout, GPR40 agonists for the treatment of type 2 diabetes: Life after 'TAKing' a hit. *Diabetes Obes. Metab.* **17**, 622–629 (2015).

10. G. Milligan, E. Alvarez-Curto, B. D. Hudson, R. Prihandoko, A. B. Tobin, FFA4/GPR120: Pharmacology and therapeutic opportunities. *Trends Pharmacol. Sci.* **38**, 809–821 (2017).

11. G. Carullo, S. Mazzotta, M. Vega-Holm, F. Iglesias-Guerra, J. M. Vega-Perez, F. Aiello, A. Brizzi, GPR120/FFAR4 pharmacology: Focus on agonists in type 2 diabetes mellitus drug discovery. *J. Med. Chem.* **64**, 4312–4332 (2021).

12. S. Halder, S. Kumar, R. Sharma, The therapeutic potential of GPR120: A patent review. *Expert Opin. Ther. Pat.* **23**, 1581–1590 (2013).

13. G. Bianchini, C. Nigro, A. Sirico, R. Novelli, I. Prevenzano, C. Miele, F. Beguinot, A. Aramini, A new synthetic dual agonist of GPR120/GPR40 induces GLP-1 secretion and improves glucose homeostasis in mice. *Biomed. Pharmacother.* **139**, 111613 (2021).

14. X. Zhang, M. J. Macielag, GPR120 agonists for the treatment of diabetes: A patent review (2014 present). *Expert Opin. Ther. Pat.* **30**, 729–742 (2020).

15. C. Cartoni, K. Yasumatsu, T. Ohkuri, N. Shigemura, R. Yoshida, N. Godinot, J. le Coutre, Y. Ninomiya, S. Damak, Taste preference for fatty acids is mediated by GPR40 and GPR120. *J. Neurosci.* **30**, 8376–8382 (2010).

16. M. H. Kim, S. G. Kang, J. H. Park, M. Yanagisawa, C. H. Kim, Short-chain fatty acids activate GPR41 and GPR43 on intestinal epithelial cells to promote inflammatory responses in mice. *Gastroenterology* **145**, 396–406.e1–10 (2013).

17. P. D. Cani, A. Everard, T. Duparc, Gut microbiota, enteroendocrine functions and metabolism. *Curr. Opin. Pharmacol.* **13**, 935–940 (2013).

18. K. M. Maslowski, A. T. Vieira, A. Ng, J. Kranich, F. Sierro, D. Yu, H. C. Schilter, M. S. Rolph, F. Mackay, D. Artis, R. J. Xavier, M. M. Teixeira, C. R. Mackay, Regulation of inflammatory responses by gut microbiota and chemoattractant receptor GPR43. *Nature* **461**, 1282–1286 (2009).

19. I. Kimura, K. Ozawa, D. Inoue, T. Imamura, K. Kimura, T. Maeda, K. Terasawa, D. Kashihara, K. Hirano, T. Tani, T. Takahashi, S. Miyauchi, G. Shioi, H. Inoue, G. Tsujimoto, The gut microbiota suppresses insulin-mediated fat accumulation via the short-chain fatty acid receptor GPR43. *Nat. Commun.* **4**, 1829 (2013).

20. Y. Zhao, F. Chen, W. Wu, M. Sun, A. J. Bilotta, S. Yao, Y. Xiao, X. Huang, T. D. Eaves-Pyles, G. Golovko, Y. Fofanov, W. D'Souza, Q. Zhao, Z. Liu, Y. Cong, GPR43 mediates microbiota metabolite SCFA regulation of antimicrobial peptide expression in intestinal epithelial cells via activation of mTOR and STAT3. *Mucosal Immunol.* **11**, 752–762 (2018).

21. K. H. Antunes, J. L. Fachi, R. de Paula, E. F. da Silva, L. P. Pral, A. A. Dos Santos, G. B. M. Dias, J. E. Vargas, R. Puga, F. Q. Mayer, F. Maito, C. R. Zarate-Blades, N. J. Ajami, M. R. Sant'Ana, T. Candreva, H. G. Rodrigues, M. Schmiele, M. T. P. Silva Clerici, J. L. Proenca-Modena, A. T. Vieira, C. R. Mackay, D. Mansur, M. T. Caballero, J. Marzec, J. Li, X. Wang, D. Bell, F. P. Polack, S. R. Kleeberger, R. T. Stein, M. A. R. Vinolo, A. P. D. de Souza, Microbiota-derived acetate protects against respiratory syncytial virus infection through a GPR43-type 1 interferon response. *Nat. Commun.* **10**, 3273 (2019).

22. L. B. Bindels, E. M. Dewulf, N. M. Delzenne, GPR43/FFA2: Physiopathological relevance and therapeutic prospects. *Trends Pharmacol. Sci.* **34**, 226–232 (2013).

23. S. Kim, Y. M. Kim, Y. S. Kwak, A novel therapeutic target, GPR43; where it stands in drug discovery. *Arch. Pharm. Res.* **35**, 1505–1509 (2012).

24. I. Kimura, D. Inoue, K. Hirano, G. Tsujimoto, The SCFA receptor GPR43 and energy metabolism. *Front. Endocrinol. (Lausanne)* **5**, 85 (2014).

25. G. Milligan, B. Shimpukade, T. Ulven, B. D. Hudson, Complex pharmacology of free fatty acid receptors. *Chem. Rev.* **117**, 67–110 (2017).

26. M. Grundmann, E. Bender, J. Schamberger, F. Eitner, Pharmacology of free fatty acid receptors and their allosteric modulators. *Int. J. Mol. Sci.* **22**, 1763 (2021).

27. K. Moore, Q. Zhang, N. Murgolo, T. Hosted, R. Duffy, Cloning, expression, and pharmacological characterization of the GPR120 free fatty acid receptor from cynomolgus monkey: Comparison with human GPR120 splice variants. *Comp. Biochem. Physiol. B Biochem. Mol. Biol.* **154**, 419–426 (2009).

28. S.-J. Watson, A. J. Brown, N. D. Holliday, Differential signaling by splice variants of the human free fatty acid receptor GPR120. *Mol. Pharmacol.* **81**, 631–642 (2012).

29. J. Lu, N. Byrne, J. Wang, G. Bricogne, F. K. Brown, H. R. Chobanian, S. L. Colletti, J. Di Salvo, B. Thomas-Fowlkes, Y. Guo, D. L. Hall, J. Hadix, N. B. Hastings, J. D. Hermes, T. Ho, A. D. Howard, H. Josien, M. Kornienko, K. J. Lumb, M. W. Miller, S. B. Patel, B. Pio, C. W. Plummer, B. S. Sherborne, P. Sheth, S. Souza, S. Tummala, C. Vonrhein, M. Webb, S. J. Allen, J. M. Johnston, A. B. Weinglass, S. Sharma, S. M. Soisson, Structural basis for the cooperative allosteric activation of the free fatty acid receptor GPR40. *Nat. Struct. Mol. Biol.* **24**, 570–577 (2017).

30. J. D. Ho, B. Chau, L. Rodgers, F. Lu, K. L. Wilbur, K. A. Otto, Y. Chen, M. Song, J. P. Riley, H. C. Yang, N. A. Reynolds, S. D. Kahl, A. P. Lewis, C. Groshong, R. E. Madsen, K. Conners, J. P. Lineswala, T. Gheyi, M. D. Saflor, M. R. Lee, J. Benach, K. A. Baker, C. Montrose-Rafizadeh, M. J. Genin, A. R. Miller, C. Hamdouchi, Structural basis for GPR40 allosteric agonism and incretin stimulation. *Nat. Commun.* **9**, 1645 (2018).

31. A. Srivastava, J. Yano, Y. Hirozane, G. Kefala, F. Gruswitz, G. Snell, W. Lane, A. Ivetac, K. Aertgeerts, J. Nguyen, A. Jennings, K. Okada, High-resolution structure of the human GPR40 receptor bound to allosteric agonist TAK-875. *Nature* **513**, 124–127 (2014).

32. K. Kim, T. Che, O. Panova, J. F. DiBerto, J. Lyu, B. E. Krumm, D. Wacker, M. J. Robertson, A. B. Seven, D. E. Nichols, B. K. Shoichet, G. Skiniotis, B. L. Roth, Structure of a hallucinogen-activated Gq-coupled 5-HT_{2A} serotonin receptor. *Cell* **182**, 1574–1588.e19 (2020).

33. R. Nehmé, B. Carpenter, A. Singhal, A. Strege, P. C. Edwards, C. F. White, H. Du, R. Grisshammer, C. G. Tate, Mini-G proteins: Novel tools for studying GPCRs in their active conformation. *PLOS ONE* **12**, e0175642 (2017).

34. B. Shimpukade, B. D. Hudson, C. K. Hovgaard, G. Milligan, T. Ulven, Discovery of a potent and selective GPR120 agonist. *J. Med. Chem.* **55**, 4511–4515 (2012).

35. Q. Wan, N. Okashah, A. Inoue, R. Nehme, B. Carpenter, C. G. Tate, N. A. Lambert, Mini G protein probes for active G protein-coupled receptors (GPCRs) in live cells. *J. Biol. Chem.* **293**, 7466–7473 (2018).

36. J. G. Meyerowitz, M. J. Robertson, X. Barros-Alvarez, O. Panova, R. M. Nwokonko, Y. Gao, G. Skiniotis, The oxytocin signaling complex reveals a molecular switch for cation dependence. *Nat. Struct. Mol. Biol.* **29**, 274–281 (2022).

37. J. Duan, D. D. Shen, X. E. Zhou, P. Bi, Q. F. Liu, Y. X. Tan, Y. W. Zhuang, H. B. Zhang, P. Y. Xu, S. J. Huang, S. S. Ma, X. H. He, K. Melcher, Y. Zhang, H. E. Xu, Y. Jiang, Cryo-EM structure of an activated VIP1 receptor-G protein complex revealed by a NanoBiT tethering strategy. *Nat. Commun.* **11**, 4121 (2020).

38. A. Koehl, H. Hu, S. Maeda, Y. Zhang, Q. Qu, J. M. Paggi, N. R. Latorraca, D. Hilger, R. Dawson, H. Matile, G. F. X. Schertler, S. Granier, W. I. Weis, R. O. Dror, A. Manglik, G. Skiniotis, B. K. Kobilka, Structure of the μ-opioid receptor-Gi protein complex. *Nature* **558**, 547–552 (2018).

39. L. Wang, D. Yao, R. Deepak, H. Liu, Q. Xiao, H. Fan, W. Gong, Z. Wei, C. Zhang, Structures of the human PGD(2) receptor CRTH2 reveal novel mechanisms for ligand recognition. *Mol. Cell* **72**, 48–59.e4 (2018).

40. H. Liu, R. Deepak, A. Shiriaeva, C. Gati, A. Batyuk, H. Hu, U. Weierstall, W. Liu, L. Wang, V. Cherezov, H. Fan, C. Zhang, Molecular basis for lipid recognition by the prostaglandin D_2 receptor CRTH2. *Proc. Natl. Acad. Sci. U.S.A.* **118**, e2102813118 (2021).

41. J. A. Ballesteros, H. Weinstein, [19] Integrated methods for the construction of three-dimensional models and computational probing of structure-function relations in G protein-coupled receptors. *J. Neurosci. Methods*, **25**, 366–428 (1995).

42. B. D. Hudson, B. Shimpukade, G. Milligan, T. Ulven, The molecular basis of ligand interaction at free fatty acid receptor 4 (FFA4/GPR120). *J. Biol. Chem.* **289**, 20345–20358 (2014).

43. G. Pandy-Szekeres, J. Caroli, A. Mamyrbekov, A. A. Kermani, G. M. Keseru, A. J. Kooistra, D. E. Gloriam, GPCRdb in 2023: State-specific structure models using AlphaFold2 and new ligand resources. *Nucleic Acids Res.* **51**, D395-D402 (2023).

44. C. M. Azevedo, K. R. Watterson, E. T. Wargent, S. V. Hansen, B. D. Hudson, M. A. Kepczynska, J. Dunlop, B. Shimpukade, E. Christiansen, G. Milligan, C. J. Stocker, T. Ulven, Non-acidic free fatty acid receptor 4 agonists with antidiabetic activity. *J. Med. Chem.* **59**, 8868–8878 (2016).

45. C. Mao, P. Xiao, X. N. Tao, J. Qin, Q. T. He, C. Zhang, S. C. Guo, Y. Q. Du, L. N. Chen, D. D. Shen, Z. S. Yang, H. Q. Zhang, S. M. Huang, Y. H. He, J. Cheng, Y. N. Zhong, P. Shang, J. Chen, D. L. Zhang, Q. L. Wang, M. X. Liu, G. Y. Li, Y. Guo, H. E. Xu, C. Wang, C. Zhang, S. Feng, X. Yu, Y. Zhang, J. P. Sun, Unsaturated bond recognition leads to biased signal in a fatty acid receptor. *Science* **380**, eadd6220 (2023).

46. M. Lückmann, M. Trauelsen, T. M. Frimurer, T. W. Schwartz, Structural basis for GPCR signaling by small polar versus large lipid metabolites-discovery of non-metabolite ligands. *Curr. Opin. Cell Biol.* **63**, 38–48 (2020).

47. H. Liu, H. R. Kim, R. Deepak, L. Wang, K. Y. Chung, H. Fan, Z. Wei, C. Zhang, Orthosteric and allosteric action of the C5a receptor antagonists. *Nat. Struct. Mol. Biol.* **25**, 472–481 (2018).

48. X. Liu, J. Kaindl, M. Korczynska, A. Stossel, D. Dengler, M. Stanek, H. Hubner, M. J. Clark, J. Mahoney, R. A. Matt, X. Xu, K. Hirata, B. K. Shoichet, R. K. Sunahara, B. K. Kobilka, P. Gmeiner, An allosteric modulator binds to a conformational hub in the β2 adrenergic receptor. *Nat. Chem. Biol.* **16**, 749–755 (2020).

49. C. Yabuki, H. Komatsu, Y. Tsujihata, R. Maeda, R. Ito, K. Matsuda-Nagasumi, K. Sakuma, K. Miyawaki, N. Kikuchi, K. Takeuchi, Y. Habata, M. Mori, A novel antidiabetic drug,

fasiglifam/TAK-875, acts as an ago-allosteric modulator of FFAR1. *PLOS ONE* **8**, e76280 (2013).

50. P. Kumari, A. Inoue, K. Chapman, P. Lian, D. M. Rosenbaum, Molecular mechanism of fatty acid activation of FFAR1. *Proc. Natl. Acad. Sci. U.S.A.* **120**, e2219569120 (2023).

51. F. Yang, C. Mao, L. Guo, J. Lin, Q. Ming, P. Xiao, X. Wu, Q. Shen, S. Guo, D. D. Shen, R. Lu, L. Zhang, S. Huang, Y. Ping, C. Zhang, C. Ma, K. Zhang, X. Liang, Y. Shen, F. Nan, F. Yi, V. C. Luca, J. Zhou, C. Jiang, J. P. Sun, X. Xie, X. Yu, Y. Zhang, Structural basis of GPBAR activation and bile acid recognition. *Nature* **587**, 499–504 (2020).

52. L. A. Stoddart, N. J. Smith, G. Milligan, International Union of Pharmacology. LXXI. Free fatty acid receptors FFA1, -2, and -3: Pharmacology and pathophysiological functions. *Pharmacol. Rev.* **60**, 405–417 (2008).

53. L. A. Stoddart, N. J. Smith, L. Jenkins, A. J. Brown, G. Milligan, Conserved polar residues in transmembrane domains V, VI, and VII of free fatty acid receptor 2 and free fatty acid receptor 3 are required for the binding and function of short chain fatty acids. *J. Biol. Chem.* **283**, 32913–32924 (2008).

54. E. Sergeev, A. H. Hansen, S. K. Pandey, A. E. MacKenzie, B. D. Hudson, T. Ulven, G. Milligan, Non-equivalence of key positively charged residues of the free fatty acid 2 receptor in the recognition and function of agonist versus antagonist ligands. *J. Biol. Chem.* **291**, 303–317 (2016).

55. B. D. Hudson, M. E. Due-Hansen, E. Christiansen, A. M. Hansen, A. E. Mackenzie, H. Murdoch, S. K. Pandey, R. J. Ward, R. Marquez, I. G. Tikhonova, T. Ulven, G. Milligan, Defining the molecular basis for the first potent and selective orthosteric agonists of the FFA2 free fatty acid receptor. *J. Biol. Chem.* **288**, 17296–17312 (2013).

56. B. D. Hudson, E. Christiansen, I. G. Tikhonova, M. Grundmann, E. Kostenis, D. R. Adams, T. Ulven, G. Milligan, Chemically engineering ligand selectivity at the free fatty acid receptor 2 based on pharmacological variation between species orthologs. *FASEB J.* **26**, 4951–4965 (2012).

57. E. Sergeev, A. H. Hansen, D. Bolognini, K. Kawakami, T. Kishi, J. Aoki, T. Ulven, A. Inoue, B. D. Hudson, G. Milligan, A single extracellular amino acid in free fatty acid receptor 2 defines antagonist species selectivity and G protein selection bias. *Sci. Rep.* **7**, 13741 (2017).

58. B. D. Hudson, I. G. Tikhonova, S. K. Pandey, T. Ulven, G. Milligan, Extracellular ionic locks determine variation in constitutive activity and ligand potency between species orthologs of the free fatty acid receptors FFA2 and FFA3. *J. Biol. Chem.* **287**, 41195–41209 (2012).

59. S. Filipek, Molecular switches in GPCRs. *Curr. Opin. Struct. Biol.* **55**, 114–120 (2019).

60. Q. Zhou, D. Yang, M. Wu, Y. Guo, W. Guo, L. Zhong, X. Cai, A. Dai, W. Jang, E. I. Shakhnovich, Z. J. Liu, R. C. Stevens, N. A. Lambert, M. M. Babu, M. W. Wang, S. Zhao, Common activation mechanism of class A GPCRs. *eLife* **8**, e50279 (2019).

61. L. Heo, M. Feig, Multi-state modeling of G-protein coupled receptors at experimental accuracy. *Proteins* **90**, 1873–1885 (2022).

62. W. I. Weis, B. K. Kobilka, The molecular basis of G protein-coupled receptor activation. *Annu. Rev. Biochem.* **87**, 897–919 (2018).

63. S. G. Rasmussen, H. J. Choi, J. J. Fung, E. Pardon, P. Casarosa, P. S. Chae, B. T. Devree, D. M. Rosenbaum, F. S. Thian, T. S. Kobilka, A. Schnapp, I. Konetzki, R. K. Sunahara, S. H. Gellman, A. Pautsch, J. Steyaert, W. I. Weis, B. K. Kobilka, Structure of a nanobody-stabilized active state of the β_2 adrenoceptor. *Nature* **469**, 175–180 (2011).

64. W. M. Oldham, H. E. Hamm, Heterotrimeric G protein activation by G-protein-coupled receptors. *Nat. Rev. Mol. Cell Biol.* **9**, 60–71 (2008).

65. S. Maeda, Q. Qu, M. J. Robertson, G. Skiniotis, B. K. Kobilka, Structures of the M1 and M2 muscarinic acetylcholine receptor/G-protein complexes. *Science* **364**, 552–557 (2019).

66. A. H. Hansen, E. Sergeev, D. Bolognini, R. R. Sprenger, J. H. Ekberg, C. S. Ejsing, C. J. McKenzie, E. Rexen Ulven, G. Milligan, T. Ulven, Discovery of a potent thiazolidine free fatty

acid receptor 2 agonist with favorable pharmacokinetic properties. *J. Med. Chem.* **61**, 9534–9550 (2018).

67. K. Kaku, K. Enya, R. Nakaya, T. Ohira, R. Matsuno, Efficacy and safety of fasiglifam (TAK-875), a G protein-coupled receptor 40 agonist, in Japanese patients with type 2 diabetes inadequately controlled by diet and exercise: A randomized, double-blind, placebo-controlled, phase III trial. *Diabetes Obes. Metab.* **17**, 675–681 (2015).

68. M. Kim, G. J. Gu, Y. S. Koh, S. H. Lee, Y. R. Na, S. H. Seok, K. M. Lim, Fasiglifam (TAK-875), a G protein-coupled receptor 40 (GPR40) agonist, may induce hepatotoxicity through reactive oxygen species generation in a GPR40-dependent manner. *Biomol. Ther. (Seoul)* **26**, 599–607 (2018).

69. M. A. Otieno, J. Snoeys, W. Lam, A. Ghosh, M. R. Player, A. Pocai, R. Salter, D. Simic, H. Skaggs, B. Singh, H. K. Lim, Fasiglifam (TAK-875): Mechanistic investigation and retrospective identification of hazards for drug induced liver injury. *Toxicol. Sci.* **163**, 374–384 (2018).

70. I. G. Tikhonova, Application of GPCR structures for modelling of free fatty acid receptors. *Handb. Exp. Pharmacol.* **236**, 57–77 (2017).

71. A. Ciancetta, A. K. Gill, T. Ding, D. S. Karlov, G. Chalhoub, P. J. McCormick, I. G. Tikhonova, Probe confined dynamic mapping for G protein-coupled receptor allosteric site prediction. *ACS Cent. Sci.* **7**, 1847–1862 (2021).

72. D. N. Mastronarde, Automated electron microscope tomography using robust prediction of specimen movements. *J. Struct. Biol.* **152**, 36–51 (2005).

73. A. Punjani, J. L. Rubinstein, D. J. Fleet, M. A. Brubaker, cryoSPARC: Algorithms for rapid unsupervised cryo-EM structure determination. *Nat. Methods* **14**, 290–296 (2017).

74. E. F. Pettersen, T. D. Goddard, C. C. Huang, G. S. Couch, D. M. Greenblatt, E. C. Meng, T. E. Ferrin, UCSF Chimera—A visualization system for exploratory research and analysis. *J. Comput. Chem.* **25**, 1605–1612 (2004).

75. P. Emsley, K. Cowtan, Coot: Model-building tools for molecular graphics. *Acta Crystallogr. D Biol. Crystallogr.* **60**, 2126–2132 (2004).

76. P. D. Adams, P. V. Afonine, G. Bunkoczi, V. B. Chen, I. W. Davis, N. Echols, J. J. Headd, L. W. Hung, G. J. Kapral, R. W. Grosse-Kunstleve, A. J. McCoy, N. W. Moriarty, R. Oeffner, R. J. Read, D. C. Richardson, J. S. Richardson, T. C. Terwilliger, P. H. Zwart, PHENIX: A comprehensive Python-based system for macromolecular structure solution. *Acta Crystallogr. D Biol. Crystallogr.* **66**, 213–221 (2010).

77. V. B. Chen, W. B. Arendall, 3rd, J. J. Headd, D. A. Keedy, R. M. Immormino, G. J. Kapral, L. W. Murray, J. S. Richardson, D. C. Richardson, MolProbity: All-atom structure validation for macromolecular crystallography. *Acta Crystallogr. D Biol. Crystallogr.* **66**, 12–21 (2010).

78. B. D. Hudson, B. Shimpukade, A. E. Mackenzie, A. J. Butcher, J. D. Pediani, E. Christiansen, H. Heathcote, A. B. Tobin, T. Ulven, G. Milligan, The pharmacology of TUG-891, a potent and selective agonist of the free fatty acid receptor 4 (FFA4/GPR120), demonstrates both potential opportunity and possible challenges to therapeutic agonism. *Mol. Pharmacol.* **84**, 710–725 (2013).

79. Schrödinger Release 2022-3: Maestro (Schrödinger LLC, 2021).

80. S. Jo, T. Kim, W. Im, Automated builder and database of protein/membrane complexes for molecular dynamics simulations. *PLOS ONE* **2**, e880 (2007).

81. S. Jo, J. B. Lim, J. B. Klauda, W. Im, CHARMM-GUI membrane builder for mixed bilayers and its application to yeast membranes. *Biophys. J.* **97**, 50–58 (2009).

82. E. L. Wu, X. Cheng, S. Jo, H. Rui, K. C. Song, E. M. Davila-Contreras, Y. Qi, J. Lee, V. Monje-Galvan, R. M. Venable, J. B. Klauda, W. Im, CHARMM-GUI membrane builder toward realistic biological membrane simulations. *J. Comput. Chem.* **35**, 1997–2004 (2014).

83. J. Lee, X. Cheng, J. M. Swails, M. S. Yeom, P. K. Eastman, J. A. Lemkul, S. Wei, J. Buckner, J. C. Jeong, Y. Qi, S. Jo, V. S. Pande, D. A. Case, C. L. Brooks, 3rd, A. D. MacKerell, Jr., J. B. Klauda, W. Im, CHARMM-GUI input generator for NAMD, GROMACS, AMBER, OpenMM,

and CHARMM/OpenMM simulations using the CHARMM36 additive force field. *J. Chem. Theory Comput.* **12**, 405–413 (2016).

84. S. Kim, J. Lee, S. Jo, C. L. Brooks, 3rd, H. S. Lee, W. Im, CHARMM-GUI ligand reader and modeler for CHARMM force field generation of small molecules. *J. Comput. Chem.* **38**, 1879–1886 (2017).

85. J. Lee, D. S. Patel, J. Stahle, S. J. Park, N. R. Kern, S. Kim, J. Lee, X. Cheng, M. A. Valvano, O. Holst, Y. A. Knirel, Y. Qi, S. Jo, J. B. Klauda, G. Widmalm, W. Im, CHARMM-GUI membrane builder for complex biological membrane simulations with glycolipids and lipoglycans. *J. Chem. Theory Comput.* **15**, 775–786 (2019).

86. J. Lee, M. Hitzenberger, M. Rieger, N. R. Kern, M. Zacharias, W. Im, CHARMM-GUI supports the amber force fields. *J. Chem. Phys.* **153**, 035103 (2020).

87. S. Jo, T. Kim, V. G. Iyer, W. Im, CHARMM-GUI: A web-based graphical user interface for CHARMM. *J. Comput. Chem.* **29**, 1859–1865 (2008).

88. B. R. Brooks, C. L. Brooks, 3rd, A. D. Mackerell, Jr., L. Nilsson, R. J. Petrella, B. Roux, Y. Won, G. Archontis, C. Bartels, S. Boresch, A. Caflisch, L. Caves, Q. Cui, A. R. Dinner, M. Feig, S. Fischer, J. Gao, M. Hodoscek, W. Im, K. Kuczera, T. Lazaridis, J. Ma, V. Ovchinnikov, E. Paci, R. W. Pastor, C. B. Post, J. Z. Pu, M. Schaefer, B. Tidor, R. M. Venable, H. L. Woodcock, X. Wu, W. Yang, D. M. York, M. Karplus, CHARMM: The biomolecular simulation program. *J. Comput. Chem.* **30**, 1545–1614 (2009).

89. R. Salomon-Ferrer, A. W. Gotz, D. Poole, S. Le Grand, R. C. Walker, Routine microsecond molecular dynamics simulations with AMBER on GPUs. 2. Explicit solvent particle mesh ewald. *J Chem Theory Comput* **9**, 3878–3888 (2013).

90. A. W. G. Scott Le Grand, Ross C. Walker, SPFP: Speed without compromise—A mixed precision model for GPU accelerated molecular dynamics simulations. *Comput. Phys. Commun.* **184**, 374–380 (2013).

91. K. B. D. A. Case, I. Y. Ben-Shalom, S. R. Brozell, D. S. Cerutti, T. E. Cheatham, III, V. W. D. Cruzeiro,, R. E. D. T. A. Darden, G. Giambasu, M. K. Gilson, H. Gohlke, A. W. Goetz,R Harris, S. Izadi, S. A. Izmailov, K. Kasavajhala, A. Kovalenko, R. Krasny, T. Kurtzman, T. S. Lee, S. LeGrand, P. Li, C. Lin, J. Liu,, R. L. T. Luchko, V. Man, K. M. Merz, Y. Miao, O. Mikhailovskii, G. Monard, H. Nguyen, A. Onufriev, F., S. P. Pan, R. Qi, D. R. Roe, A. Roitberg, *C. Sagui*, S. Schott-Verdugo, J. Shen, C. L. Simmerling, N. R., J. S. Skrynnikov, J. Swails, R. C. Walker, J. Wang, L. Wilson, R. M. Wolf, X. Wu, Y. Xiong, Y. Xue,, P. A. K. D. M. York, *AMBER 2020* (University of California, San Francisco, 2020).

92. N. Michaud-Agrawal, E. J. Denning, T. B. Woolf, O. Beckstein, MDAnalysis: A toolkit for the analysis of molecular dynamics simulations. *J. Comput. Chem.* **32**, 2319–2327 (2011).

93. R. Gowers, M. Linke, J. Barnoud, T. Reddy, M. Melo, S. Seyler, J. Domański, D. Dotson, S. Buchoux, I. Kenney, O. Beckstein, in *Proceedings of the 15th Python in Science Conference* (2016), pp. 98–105.

94. W. Humphrey, A. Dalke, K. Schulten, VMD: Visual molecular dynamics. *J. Mol. Graph.* **14**, 27–38 (1996).

95. J. C. Phillips, R. Braun, W. Wang, J. Gumbart, E. Tajkhorshid, E. Villa, C. Chipot, R. D. Skeel, L. Kale, K. Schulten, Scalable molecular dynamics with NAMD. *J. Comput. Chem.* **26**, 1781–1802 (2005).

96. J. C. Phillips, D. J. Hardy, J. D. C. Maia, J. E. Stone, J. V. Ribeiro, R. C. Bernardi, R. Buch, G. Fiorin, J. Henin, W. Jiang, R. McGreevy, M. C. R. Melo, B. K. Radak, R. D. Skeel, A. Singharoy, Y. Wang, B. Roux, A. Aksimentiev, Z. Luthey-Schulten, L. V. Kale, K. Schulten, C. Chipot, E. Tajkhorshid, Scalable molecular dynamics on CPU and GPU architectures with NAMD. *J. Chem. Phys.* **153**, 044130 (2020).

97. G. Pandy-Szekeres, C. Munk, T. M. Tsonkov, S. Mordalski, K. Harpsoe, A. S. Hauser, A. J. Bojarski, D. E. Gloriam, GPCRdb in 2018: Adding GPCR structure models and ligands. *Nucleic Acids Res.* **46**, D440–D446 (2018).

98. A. Rambaut (Institute of Evolutionary Biology, University of Edinburgh, 2018).

99. R. A. Friesner, J. L. Banks, R. B. Murphy, T. A. Halgren, J. J. Klicic, D. T. Mainz, M. P. Repasky, E. H. Knoll, M. Shelley, J. K. Perry, D. E. Shaw, P. Francis, P. S. Shenkin, Glide: A new approach for rapid, accurate docking and scoring. 1. Method and assessment of docking accuracy. *J. Med. Chem.* **47**, 1739–1749 (2004).

100. T. A. Halgren, R. B. Murphy, R. A. Friesner, H. S. Beard, L. L. Frye, W. T. Pollard, J. L. Banks, Glide: A new approach for rapid, accurate docking and scoring. 2. Enrichment factors in database screening. *J. Med. Chem.* **47**, 1750–1759 (2004).

101. R. A. Friesner, R. B. Murphy, M. P. Repasky, L. L. Frye, J. R. Greenwood, T. A. Halgren, P. C. Sanschagrin, D. T. Mainz, Extra precision glide: Docking and scoring incorporating a model of hydrophobic enclosure for protein-ligand complexes. *J. Med. Chem.* **49**, 6177–6196 (2006).

102. Glide (Schrödinger LLC, 2021).

103. Prime (Schrödinger LLC, 2021).

104. M. P. Jacobson, D. L. Pincus, C. S. Rapp, T. J. Day, B. Honig, D. E. Shaw, R. A. Friesner, A hierarchical approach to all-atom protein loop prediction. *Proteins* **55**, 351–367 (2004).

105. X. He, V. H. Man, B. Ji, X. Q. Xie, J. Wang, Calculate protein-ligand binding affinities with the extended linear interaction energy method: Application on the Cathepsin S set in the D3R Grand Challenge 3. *J. Comput. Aided Mol. Des.* **33**, 105–117 (2019).

106. C. UniProt, UniProt: The Universal Protein Knowledgebase in 2023. *Nucleic Acids Res.* **51**, D523–D531 (2023).

107. M. H. Olsson, C. R. Sondergaard, M. Rostkowski, J. H. Jensen, PROPKA3: Consistent treatment of internal and surface residues in empirical pKa predictions. *J. Chem. Theory Comput.* **7**, 525–537 (2011).

108. C. R. Søndergaard, M. H. Olsson, M. Rostkowski, J. H. Jensen, Improved treatment of ligands and coupling effects in empirical calculation and rationalization of pKa values. *J. Chem. Theory Comput.* **7**, 2284–2295 (2011).

109. M. A. Lomize, I. D. Pogozheva, H. Joo, H. I. Mosberg, A. L. Lomize, OPM database and PPM web server: Resources for positioning of proteins in membranes. *Nucleic Acids Res.* **40**, D370–D376 (2012).

110. C. Tian, K. Kasavajhala, K. A. A. Belfon, L. Raguette, H. Huang, A. N. Migues, J. Bickel, Y. Wang, J. Pincay, Q. Wu, C. Simmerling, ff19SB: Amino-acid-specific protein backbone parameters trained against quantum mechanics energy surfaces in solution. *J. Chem. Theory Comput.* **16**, 528–552 (2020).

111. C. J. Dickson, R. C. Walker, I. R. Gould, Lipid21: Complex lipid membrane simulations with amber. *J. Chem. Theory Comput.* **18**, 1726–1736 (2022).

112. X. He, V. H. Man, W. Yang, T. S. Lee, J. Wang, A fast and high-quality charge model for the next generation general AMBER force field. *J. Chem. Phys.* **153**, 114502 (2020).

113. W. L. Jorgensen, J. Chandrasekhar, J. D. Madura, R. W. Impey, M. L. Klein, Comparison of simple potential functions for simulating liquid water. *J. Chem. Phys.* **79**, 926–935 (1983).

114. H. J. C. Berendsen, J. P. M. Postma, W. F. Vangunsteren, A. Dinola, J. R. Haak, Molecular-dynamics with coupling to an external bath. *J. Chem. Phys.* **81**, 3684–3690 (1984).

115. R. W. Pastor, B. R. Brooks, A. Szabo, An analysis of the accuracy of Langevin and molecular dynamics algorithms. *Mol. Phys.* **65**, 1409–1419 (1988).
